# Distributed Cerebellar Motor Learning: A Spike-Timing-Dependent Plasticity Model

**DOI:** 10.3389/fncom.2016.00017

**Published:** 2016-03-02

**Authors:** Niceto R. Luque, Jesús A. Garrido, Francisco Naveros, Richard R. Carrillo, Egidio D'Angelo, Eduardo Ros

**Affiliations:** ^1^Department of Computer Architecture and Technology, Research Centre for Information and Communications Technologies of the University of Granada (CITIC-UGR)Granada, Spain; ^2^Brain Connectivity Center, Istituto di Ricovero e Cura a Carattere Scientifico, Istituto Neurologico Nazionale Casimiro MondinoPavia, Italy; ^3^Department of Brain and Behavioural Sciences, University of PaviaPavia, Italy

**Keywords:** cerebellar nuclei, spike-timing-dependent plasticity, motor learning consolidation, cerebellar modeling, cerebellar motor control

## Abstract

Deep cerebellar nuclei neurons receive both inhibitory (GABAergic) synaptic currents from Purkinje cells (within the cerebellar cortex) and excitatory (glutamatergic) synaptic currents from mossy fibers. Those two deep cerebellar nucleus inputs are thought to be also adaptive, embedding interesting properties in the framework of accurate movements. We show that distributed spike-timing-dependent plasticity mechanisms (STDP) located at different cerebellar sites (parallel fibers to Purkinje cells, mossy fibers to deep cerebellar nucleus cells, and Purkinje cells to deep cerebellar nucleus cells) in close-loop simulations provide an explanation for the complex learning properties of the cerebellum in motor learning. Concretely, we propose a new mechanistic cerebellar spiking model. In this new model, deep cerebellar nuclei embed a dual functionality: deep cerebellar nuclei acting as a gain adaptation mechanism and as a facilitator for the slow memory consolidation at mossy fibers to deep cerebellar nucleus synapses. Equipping the cerebellum with excitatory (e-STDP) and inhibitory (i-STDP) mechanisms at deep cerebellar nuclei afferents allows the accommodation of synaptic memories that were formed at parallel fibers to Purkinje cells synapses and then transferred to mossy fibers to deep cerebellar nucleus synapses. These adaptive mechanisms also contribute to modulate the deep-cerebellar-nucleus-output firing rate (output gain modulation toward optimizing its working range).

## Introduction

Since Marr (1969) and Albus (1971), the cerebellar loop has been extensively modeled providing smart explanations on how the forward-controller operations in biological systems seem to work. The classic long-term synaptic plasticity between parallel fibers (PF) and Purkinje cells (PC) [driven by the inferior olive (IO) action] stands at the core of those processes related to sensorimotor adaptation and motor control. However, this adaptation mechanism can be enhanced with complementary plasticity sites at the cerebellar circuit. Particularly, in this work we explore how STDP at Deep Cerebellar Nuclei efficiently complements the classical PF–PC long-term plasticity as an efficient adaptive gain term and memory consolidation resource.

### Plasticity in deep cerebellar nuclei

It is worth revisiting the original theories based on the structural analysis of cerebellar connectivity (Eccles, 1967; Eccles et al., 1967; Marr, 1969; Albus, 1971; Fujita, 1982). In those theories, the cerebellum was proposed to act as a timing and learning machine. The granular layer was hypothesized to recode the input spatiotemporal activity into sparse somatosensory activity. Then, only the relevant patterns were learnt and stored at PF–PC synapses under the supervised control of the teaching signal supplied by climbing fibers (CF). In light of different electrophysiological findings, it has been suggested that the CFs convey sensory feedback from comparing proprioceptive and predicted signals. CFs could indeed provide quantitative error estimation (Bazzigaluppi et al., 2012; De Gruijl et al., 2012) that, in turn, would be able to improve motor performance through specifically depressing the PF (PF–LTD) synapses that are more correlated to motor errors.

Although since the early 70s, plasticity in the cerebellar cortex was widely accepted and established, demonstrations of synaptic plasticity in cerebellar learning at cerebellar nucleus cells were studied significantly later. It was at the end of the 1990s when the analysis of the circuit-cerebellar basis for learning eye-movement yielded insight into a plausible two-state learning mechanism (Shadmehr and Brashers-Krug, 1997; Shadmehr and Holcomb, 1997). That is, whilst a fast learning process occurs in the cerebellar cortex (granular and molecular layer, involving PF–PC plasticity), a slow consolidation process occurs in deeper structures (possibly, at the deep cerebellar nuclei, DCN; Shadmehr and Brashers-Krug, 1997; Shadmehr and Holcomb, 1997; Medina and Mauk, 2000; Ohyama et al., 2006).

The main idea behind this speculative scheme lies on assuming that PF and PC outcomes are mediated by upstream-processing-nervous centers and, in turn, PC outcome shapes the output of its corresponding DCN-target neurons (Miles and Lisberger, 1981; Zhang and Linden, 2006; Zheng and Raman, 2010). This two-state learning mechanism was motivated by the fact that DCN neurons are innervated by excitatory synapses from mossy fibers (MFs) as well as by inhibitory synapses from PCs. The interplay between these excitatory and inhibitory connections has not been well-established yet. However, evidence of synaptic-plasticity traces at MFs (Racine et al., 1986; Medina and Mauk, 1999; Ohyama et al., 2006; Pugh and Raman, 2006; Zhang and Linden, 2006; Yang and Lisberger, 2014) and at PC synapses (Morishita and Sastry, 1996; Aizenman et al., 1998; Ouardouz and Sastry, 2000; Masuda and Amari, 2008) in the cerebellar nuclei and their vestibular nucleus (VN) counterparts has recently been encountered. This motivates the development of an adequate mechanistic model toward better understanding the potential of the DCN plasticity role.

Deep nucleus plasticity is assumed to be supervised and, according to different hypotheses, it is thought to be responsible for storing granular layer patterns that are correlated with the teaching signal generated by PCs (Hansel et al., 2001; Boyden et al., 2004; Gao et al., 2012). This plasticity comprises several mechanisms generating LTP and LTD at MF–DCN (Bagnall and du Lac, 2006; Pugh and Raman, 2006) and PC–DCN synapses (Morishita and Sastry, 1996; Aizenman et al., 1998; Ouardouz and Sastry, 2000). MF–DCN and PF–DCN plasticity are indeed thought to be important in controlling cerebellar learning in the context of the eye-blink classic conditioning (EBCC; Medina and Mauk, 1999, 2000). The equivalent forms of plasticity in the VN are also important in controlling cerebellar learning in the vestibulo-ocular reflex (VOR; Masuda and Amari, 2008).

Recent works based on a simplistic cerebellar model have proposed that the MF–DCN and PC–DCN synaptic plasticity mechanisms are an adaptive cerebellar-gain control (Garrido et al., 2013a; Luque et al., 2014b). Nevertheless, those works were focused just on the functional role of these DCN learning rules, without answering the question of how these learning rules may take place as STDP mechanisms. Two main issues were addressed within these computational approaches:

- Firstly, the proposed adaptive gain controller (Garrido et al., 2013a; Luque et al., 2014b) at the cerebellum was equipped with suitable learning and memory mechanisms whose nature is still under debate (Carey, 2011; Yang and Lisberger, 2014).
- Secondly, the gain-control system involving the cerebellum was capable of optimizing its performance within wider operative ranges; concretely, keeping PF–PC adaptation mechanisms within their optimal working range.

Conversely, these approaches still lack two key features that are addressed in the present work in a more realistic and biologically plausible scenario:

- Whilst MF–DCN and PC–DCN plasticity played a key role in generating the gain controller, the way through which the slow learning consolidation process occurred was still missing. The level of detail of those previous computational approaches prevented this feature from being properly addressed.
- It was not clear how to implement the analog conceptual model of these previous approaches into a spiking-based model compatible with spiking signal processing and then endowed with long-term spike-timing-dependent plasticity mechanisms.

**Figure 1:**
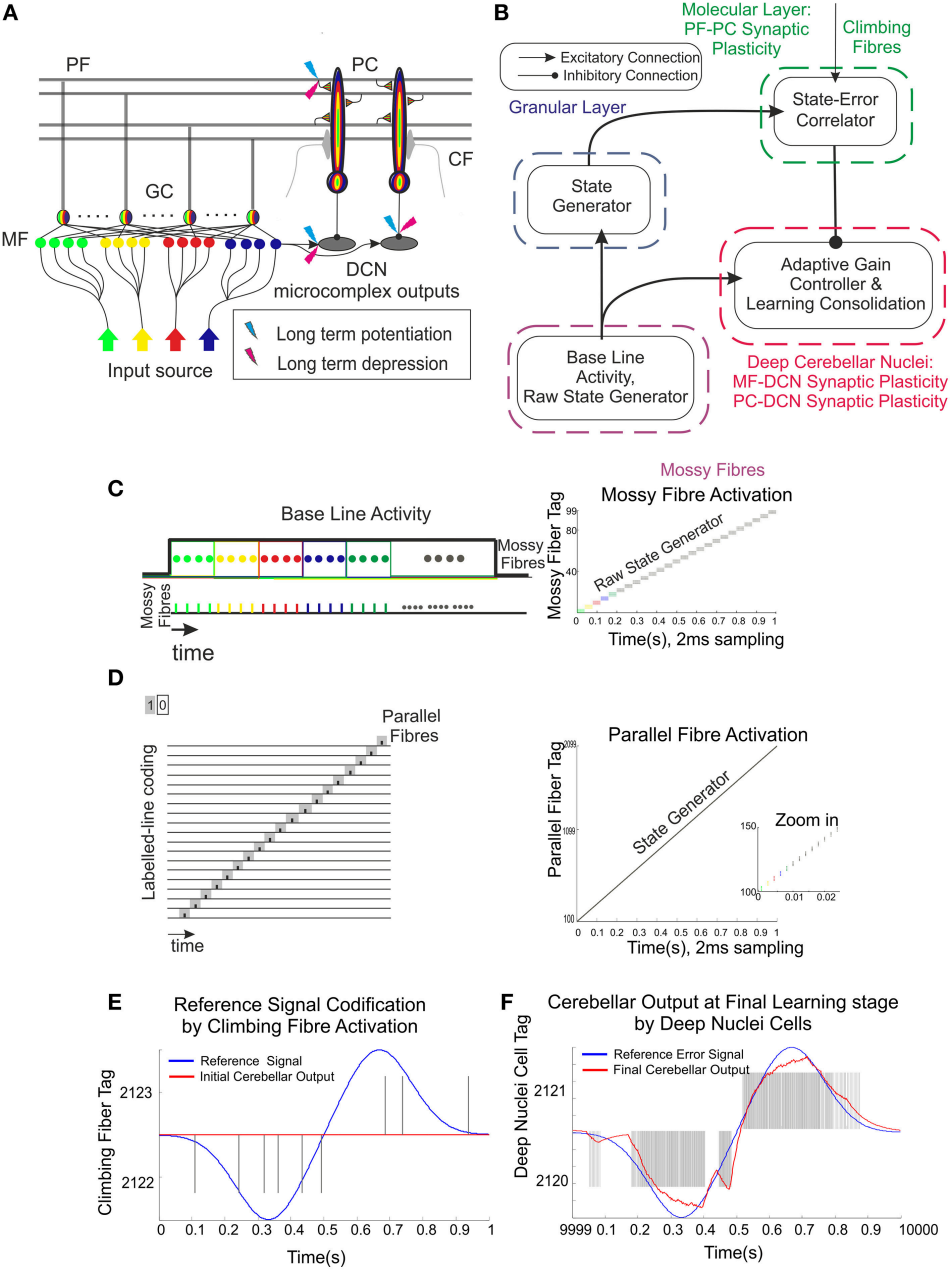
**Schematic representation of the main cerebellar layers, cerebellar cells and connections, as well as plasticity sites considered**. Working hypothesis of cerebellar learning in a manipulation task. **(A)** Cerebellar architecture. Colored representation indicates signals from different sources such as different cuneate receptive fields or proprioceptors. Pathways involved in long-term synaptic plasticity for DCN and PC afferents are indicated with two colored symbols; long-term potentiation in blue and long-term depression in magenta. PF, parallel fiber; MF, mossy fiber; CF, climbing fiber; GC, granule cell; PC, Purkinje cell; DCN, deep cerebellar nuclei. **(B)** Conceptual cerebellar block-diagram. Each cerebellar layer is put in relation to its functionality according to the cerebellar model hypothesis being adopted. MF input layer conveys sequences of spikes acting as time-evolving states (raw state generator) which present a constant firing rate, thus supplying the excitatory activity required by the DCN to start operating. The cerebellar granular layer operates as a state generator that is reinitialized with the onset of a new trial. The PC function acts as a state-error correlator; each state is correlated with the error signal that reaches the PCs through the CFs and represents the difference between the controlled variable (actual cerebellar output value) and the reference variable (set point). By repeating pairings of PF states and CF error signals, trial after trial, an association between these two sets is formed thanks to the PF–PC long-term plasticity action driven by the activity at CFs (supervised learning). A learnt corrective action is therefore deployed to anticipate the incoming error. This association implies either a reduction or increase of PC firing at different step times. Finally, the temporally correlated signals from PCs are inverted (due to the inhibitory nature of the PC–DCN connection) and conveyed to the DCN which, in turn, receives inputs coming from MF afferents (excitatory). The DCN operates like an adder/subtractor able to adaptively modulate the output DCN gain which enables learning consolidation (adapted from Garrido et al., 2013a). **(C)** During each manipulation trial, the onset of the movement makes MFs convey sequences of spikes that present a constant firing rate and time-evolving states simultaneously. This MF constant firing-rate initialization, in turn, allows PFs to start generating a non-recurrent sequence of firing states (Yamazaki and Tanaka, 2007b, 2009). To that aim, groups of non-overlapped MFs are correlatively activated during the simulation. Each colored MF group represents a certain state able to determine univocally a certain time-period within the simulation **(D)** The figure presents the GC coding strategy: in our model, the states correspond directly to non-overlapped GCs activated at each time-step simulation. Each PF group represents a certain state able to determine univocally a certain simulation step-time within the simulation **(E)** Each CF carries the teaching spikes. CF cell response follows a probabilistic Poisson process. A single spike reports time-information regarding the instantaneous error and the probabilistic spike sampling of the error ensures that the whole error region is accurately represented over trials **(F)** The generated DCN spike train is translated into meaningful analog output signals by using a Finite Impulse Response filter (FIR).

Now, in this work, we have studied the impact of distributed cerebellar spike-time synaptic plasticity on both gain adaptation and learning consolidation when performing a manipulating task. To that purpose, we have used a cerebellar spiking-based model embedded in closed loops. The working hypothesis assumes that there exist three learning sites; one located in the cerebellar cortex (PF–PC) and the other two located at the DCN innervations (MF–DCN and PC–DCN), all including LTP and LTD (Figure 1A). We found that our simulations captured the adaptive features proposed in the analog models regarding self-adaptive-gain control recalibration over a broad dynamic range involving manipulation of a heavy mass. Furthermore, we confirmed how MF–DCN innervations broadly stored what was already learnt at PF–PC. PC–DCN was also revealed as a fundamental plasticity site in charge of adapting the DCN-output firing rate.

## Materials and methods

Within this section, the working principles of the proposed mechanistic spiking cerebellar model are described. Furthermore, the major functional hypotheses related to the granular layer, PC layer, and DCN are linked to the cerebellar underlying structure (Figure 1B). The section is also divided into two main blocks. The first block describes the cerebellar topology to be used and the implemented spike-timing-dependent plasticity mechanisms. The second block consists of two case studies: Case study A uses a simplified cerebellar control loop seeking to reveal the functional interplay amongst distributed plasticity cerebellar sites, whereas Case study B uses a cerebellar control loop designed to operate a simulated robotic arm (able to manipulate heavy masses) that can exploit the potential of using distributed cerebellar plasticity in quantitative and qualitative evaluation experiments.

### Cerebellar computational model considerations

A cerebellar spiking model was implemented using the EDLUT simulator (http://edlut.googlecode.com; Ros et al., 2006; Naveros et al., 2015). This model intended to capture the essence of the main properties of synaptic cerebellar topology and its neuronal elements. This work aimed to investigate the synaptic-weight plasticity at multiple connections. The simulations were done using leaky integrate-and-fire (LIF) neural models whereas synapses were simplified using conductance based exponential models (Gerstner and Kistler, 2002). The work was focused on the IO–PC–DCN subcircuit, thus the granular layer was also simplified. That is, the granular layer was implemented as a state generator following the liquid-state-machine principles (Yamazaki and Tanaka, 2007a, 2009; see the Cerebellar-Network Organization Section). All the implemented code is at the disposal of the reader at http://www.ugr.es/~nluque/restringido/CODE.rar (user: REVIEWER, password REVIEWER).

### Cerebellar network organization

The connectivity and topology of the cerebellar network sought to abstract the general cerebellar principles taking inspiration from Eccles et al. (1967), Ito (1984), Voogd and Glickstein (1998) and Medina and Mauk (1999, 2000). Our cerebellar model consisted of four main layers (Figure 1A) connected as indicated in Table 1:

- ***Mossy fibers (MFs):*** (100) MFs were modeled as leaky I&F neurons. According to existing models of eyelid-conditioning cerebellar control (Medina and Mauk, 1999; Yamazaki and Tanaka, 2007b, 2009), MFs are hypothesized to convey sequences of spikes which present a constant firing rate during the conditioned-stimulus-presentation phase. In our simplified model, MFs were correlatively activated in non-overlapped and equally-sized neural clusters ensuring a constant firing rate during the execution of each learning trial whilst they remained silent when the learning trial came to its end. The learning trial start was defined by the onset of MF activity thus forcing the granular layer to generate its state sequence, and supplying the base-line excitatory activity that DCN needed to start operating (Figure 1C).
- ***Granular cells (GCs):*** (2000) similarly to other models (Yamazaki and Tanaka, 2005, 2007a, 2009; Honda et al., 2011), the granular layer was implemented as a state generator, that is, the granular layer generated a sequence of active neuron populations without recurrence. The sequential activation of these neuron populations was able to represent the passage of time. When the learning process began, the granular layer produced non-overlapped time patterns that were repeatedly activated in the same sequence during each learning trial (1 s; Figures 1C,D). Having 1 s learning process in a 2 ms time-step simulation demanded 500 different states, which involved four non-overlapped GCs activated per time-step simulation. PF–PC synaptic conductances were set to an initial value (5 nS) at the beginning of the simulation, and were modified by the STDP mechanism during the training process. Note that the whole model aims to adopt cell realistic ratios, although the actual number of simulated neurons is much smaller than a full size rat model. A reduced version of the cerebellum (2000 GCs) where each PC just received activity from 2000 PFs was modeled. Since in a full model of the cerebellum, each PC should receive activity from about 150,000 PFs (Brunel et al., 2004), PF–PC weight values were scaled to obtain a similar relative PC excitation.
- ***Purkinje Cells (PCs):*** We have defined two case studies: (20) Purkinje cells in case study A, (60) Purkinje cells in case study B.
  *Case study A*; the cerebellar circuit was modeled as a closed loop able to supply a corrective signal to counterbalance the existing difference between a controlled variable (actual cerebellar output value) and a demanding reference variable (set point). This was equivalent to a cerebellar model compensating the error that one degree-of-freedom (DoF) manipulator could undergo (see Control Loop Section and **Figure 3A**). Within this loop, 20 PCs inhibited two DCNs that, in turn, counterbalanced the error curve. CFs (2) were assumed to transmit the difference between the set point curve and the actual one. CFs closed the loop providing input to the PCs. This layer was divided into two groups of 10 Purkinje cells each all receiving activity through each granular layer cell. One group was in charge of correcting the negative errors (providing activity toward enhancing output cerebellar corrective activity) and the other group was in charge of corrective positive errors. This set up mimics the existing interplay between agonist and antagonist muscles at biological systems. Each 10 PC group was innervated by its corresponding CF that, in turn, was also in charge of carrying the teaching signal corresponding to the negative or the positive part of the error being estimated. Every subgroup of PCs finally inhibited a cell of the DCN that, again, counterbalanced the negative or the positive part of the error curve.
  *Case study B*; the cerebellar circuit was modeled within a closed loop designed to operate a simulated robotic arm of 3 DoFs (see Control Loop Section and **Figure 3B**). In this set up, we scaled up the cerebellar model. Within this loop, 60 PCs inhibited six DCN that, in turn, counterbalanced the error undergone by the simulated robotic arm. CFs (6) were assumed to transmit the difference between the simulated robotic arm desired-trajectory curves and the actual ones. CFs closed the loop providing teaching input to the PCs. The PC layer was divided into three groups of 20 PC cells each that were in charge of correcting their corresponding simulated robotic arm DoF. Each group was also subdivided into two groups of 10 Purkinje cells and innervated by each granular layer cell. Each subgroup of the PCs was aimed to provide the positive or negative necessary corrections. Each PC subgroup was innervated by its corresponding CF which, in turn, carried the teaching signal corresponding to either the negative or the positive part of the actual error at each DoF. Every group of PCs finally inhibited a cell of the DCN that, again, counterbalanced the negative or the positive part of the actual error.
- ***Climbing fibers (CFs):*** (2) Climbing fibers in case study A. (6) Climbing fibers in case study B. Each CF carried the teaching spikes (obtained from error signals) from the IO to a PC subgroup. CF cell response followed a probabilistic Poisson process. Given the normalized error signal ε(*t*) and a random number η(*t*) between 0 and 1, the cell fired a spike if ε(*t*) > η(*t*); otherwise, it remained silent (Boucheny et al., 2005; Luque et al., 2011a). In this way, a single spike reported accurately timed information regarding the instantaneous error; furthermore, the probabilistic spike sampling of the error ensured that the whole error region was accurately represented over trials with a constrained CF activity below 10 spikes per second, per fiber. Hence, the error evolution is accurately sampled even at a low frequency (Carrillo et al., 2008; Luque et al., 2011a). This firing behavior is similar to the ones obtained in physiological recordings (Kuroda et al., 2001; Figure 1E).
- ***Deep Cerebellar Nuclei (DCN):*** (2) Deep Cerebellar Nucleus cells in case study A, (6) Deep Cerebellar Nucleus cells in case study B. The generated DCN spike train is translated into meaningful analog output signals by using a Finite Impulse Response filter (FIR). We adopted this mathematical approach (Schrauwen and van Campenhout, 2003) because we assumed, at this stage, that the goal is to decode rather than to analyze the behavior of biological neurons.
  Defining the spike train as $x\left(t\right)=\overset{N}{\underset{j=t}{\sum }}\delta \left(t-t_{j}\right)$, where *t*_*j*_ stands for the set of firing times of the corresponding neuron, *N* is the number of events in the spike train, and being the FIR response defined as *h(t)*, then the stimulus can be written as follows (Equation 1):
  (1) $$stimulus\left(t\right)=\left(h*x\right)\left(t\right)=\sum_{j\;=\;t}^{N}h\left(t-t_{j}\right)\;j=1\;to\;N$$
  Despite the widespread use of FIR filters for such purpose, an undesired delay is introduced in the generated analog signal. This delay is strongly related to the number of filter coefficients and to the shape of the filter kernel. In order to mitigate this effect and to make the conversion more efficient, an exponentially-decaying kernel is implemented Equation (2). At each time step, the output signal value only depends on its previous value and on the input spikes in the same time step and, therefore, this filter is implemented by recursively updating the last value of the output signal. Actually, the choice of such exponential kernel is double folded. The kernel is able to mitigate the delay problem and bears a strong resemblance to postsynaptic currents (van Rossum, 2001; Victor, 2005), thus facilitating a biological interpretation. Furthermore, as demonstrated in Luque et al. (2014a), this FIR filter is equivalent to an integrative neuron (Figure 1F).
  (2) $$Kernel=e^{-\frac{M}{\tau }},\text{ where }M=1$$
  where *M* is the number of filter taps (one-tap per integration step 0.002 s) and τ is the decaying factor.
  In case study A, the cerebellum output was generated by a single group of these DCN cells; one of the cells handled positive error corrections whereas the other one handled negative error corrections. Each DCN neuron received excitation from every MF and inhibition from its corresponding 10 PC group. In this way, the sub-circuit PC–DCN–IO was organized in a single microzone.
  In case study B, the cerebellum output was generated by three groups of these cells. The cerebellar corrective output (torque) for each DoF was encoded by a group of these cells (two subgroups per DoF) whose activity provided corrective actions to the specified robot-arm commands. Each neuron group in the DCN received excitation from every MF and inhibition from its corresponding PC group. In this way, the sub-circuit PC–DCN–IO was organized in three microzones.
  In both cases, DCN synaptic conductances were set to initial values of 0 nS at the beginning of the simulation, and were modified by the STDP mechanisms during the training process.

**Table 1:** **Summary of cerebellar cells and synapses implemented in Case A and Case B simulations**.

**Case A**			**Case B**		
**Presynaptic cell (number)**	**Postsynaptic cell**	**Number of synapses**	**Presynaptic cell (number)**	**Postsynaptic cell**	**Number of synapses**
Mossy Fibers(100)	Granular Cells	8000	Mossy Fibers(100)	Granular Cells	8000
	Deep Cerebellar Nuclei	200		Deep Cerebellar Nuclei	600
Climbing Fibers(2)	Purkinje Cells	20	Climbing Fibers(6)	Purkinje Cells	60
Granular Cells(1000)	Purkinje Cells	40,000	Granular Cells(1000)	Purkinje Cells	120,000
Purkinje cell(20)	Deep Cerebellar Nuclei	20	Purkinje cell(60)	Deep Cerebellar Nuclei	60
Deep Cerebellar Nuclei(2)	–	–	Deep Cerebellar Nuclei(6)	–	–

### Synaptic plasticity

The impact of distributed cerebellar synaptic plasticity on gain adaptation and learning consolidation using close-loop experiments has been explored. It has been assumed that there are at least three learning sites, one in the cerebellar cortex (PF–PC) and two at the DCN (MF–DCN and PC–DCN), all of them generating LTP and/or LTD. Unlike the previous analog cerebellar model (Garrido et al., 2013a; Luque et al., 2014b), where each cerebellar layer was implemented as a set of parameter values corresponding to the firing rate of the neural population, the spiking model presented here preserves the timing information of the elicited spikes at each cerebellar layer and the adaptation mechanisms are based on Spike Time Dependent Synaptic Plasticity (STDP). Now we summarize these multiple forms of synaptic plasticity.

#### PF–PC synaptic plasticity

**Figure 2:**
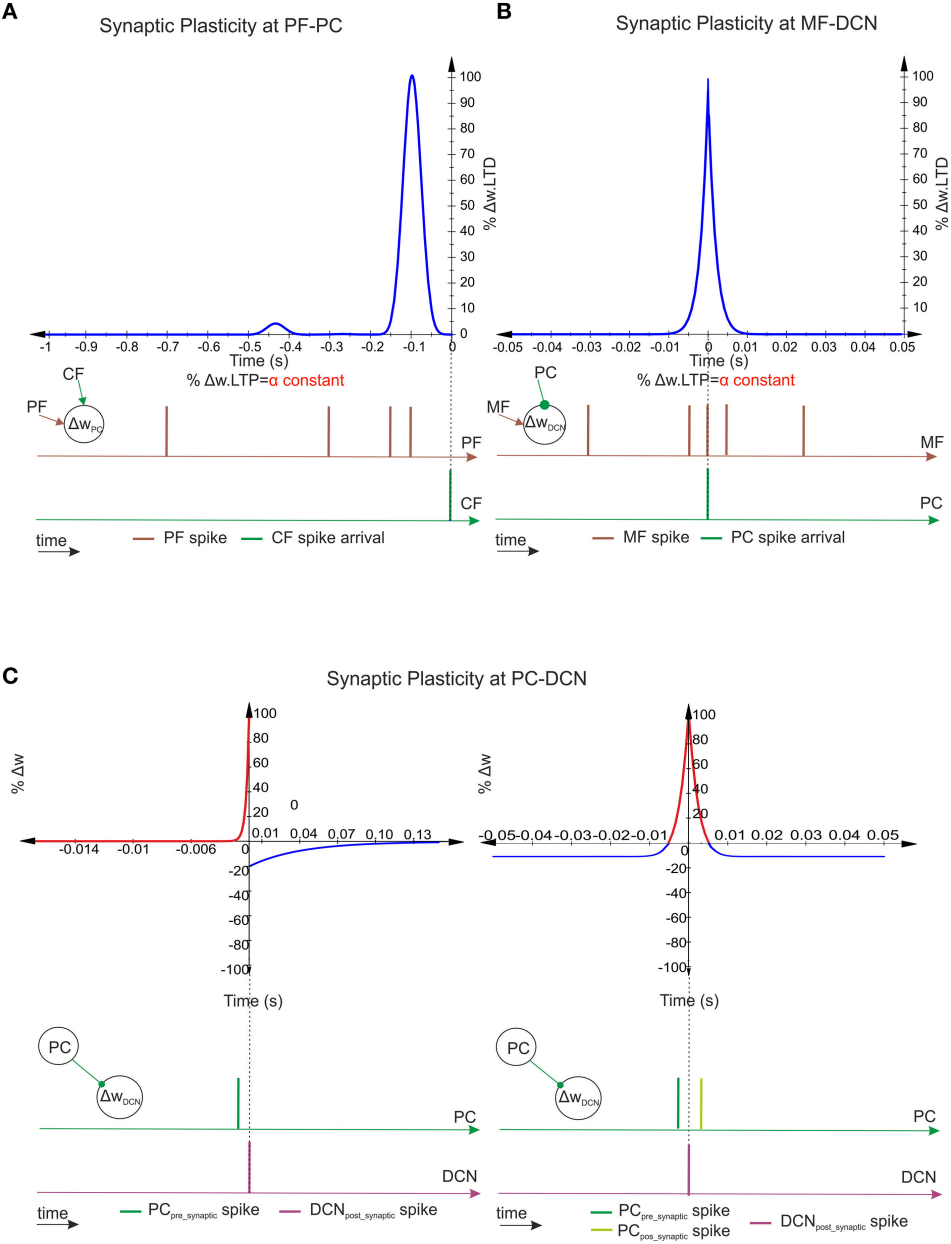
**Spike-timing–dependent learning rules at PF–PC, MF–DCN, and PC–DCN synapses. (A)** Representation of PF–PC LTD correlation kernel. A synaptic efficacy decrease occurs at PF–PC innervations when a spike from the IO reaches a target PC through a CF. The weight decrement percentage depends on the previous activity arrived through the corresponding PF (100 ms before the CF spike arrival) in order to compensate the sensorimotor pathway. The PF–PC LTP—synaptic-efficacy increase is considered to remain constant. **(B)** Representation of MF–DCN LTD correlation kernel. A synaptic efficacy decrease occurs at MF–DCN innervations when a spike from the PC reaches a target DCN. Near-coincident pre- and post-synaptic MF–DCN spikes which arrive close to PC–DCN spike arrival cause a depression at MF excitatory synapses. MF–DCN LTP-synaptic-efficacy increase is also considered to remain constant. **(C)** Representation of two PC–DCN alternative correlation kernels. Classical inhibitory STDP modifies the synapse efficacy at PC–DCN innervations depending on DCN activity. Near-coincident pre-synaptic PC–DCN spikes before post-synaptic DCN-action potentials cause long-term potentiation action whereas PC–DCN spike arrivals after post-synaptic DCN-action potentials cause long-term depression action. The second inhibitory-STDP kernel potentiates the synapse efficacy at PC–DCN innervations after a DCN-action potential each time a near-coincident pre- and postsynaptic PC-spike arrives whereas every presynaptic PC spike leads to synaptic depression.

This is, by far, the most widely investigated cerebellar plasticity mechanism as evidenced by the vast number of studies supporting the existence of multiple forms of LTD (Ito and Kano, 1982; Boyden et al., 2004; Coesmans et al., 2004) and LTP (Hansel et al., 2001; Boyden et al., 2004; Coesmans et al., 2004) plasticity mechanisms. Two important features were considered when implementing this synaptic plasticity mechanism:

- The synaptic efficacy change for each PF connection had to be driven by pre-synaptic activity (spike-timing-dependent plasticity) and had to be instantaneous.
- Since the sensorimotor pathway delay is roughly ~100 ms, the learning mechanism had to learn to provide *corrective predictions* to compensate this inner sensorimotor delay (Figure 2A).

To this aim, this plasticity mechanism was implemented including LTD and LTP as follows (Luque et al., 2011a):

- LTD produced a synaptic efficacy decrease when a spike from the IO reached the target PC through the CF. The amount of the weight decrement depended on the previous activity arrived through the PF. This previous activity was convolved with an integrative kernel as defined by Equation (3).
  (3) $$k\left(x\right)=e^{-x}\;\cdot \;\sin{\left(x\right)}^{20}$$
  where *x* is used as intermediate variable to get a compacted definition of the kernel, *x* is then substituted in Equation (4) by the independent variable *t*.
  This mainly took into account those PF spikes which arrived 100 ms before the CF spike arrival. This correction was facilitated by a time-logged “eligibility trace,” which evaluated the past activity of the afferent PF (Sutton and Barto, 1981; Barto et al., 1983; Kettner et al., 1997; Boucheny et al., 2005). This trace aimed to calculate the correspondence in time between spikes from the IO (error-related activity) and the previous activity of the PF that was temporally correlated to this error signal. The eligibility trace idea stemmed from experimental evidence showing that a spike in the climbing fiber afferent to a Purkinje cell was more likely to depress a PF–PC synapse if the corresponding PF had been firing between 50 and 150 ms before the IO spike (through CF) arrived at the PC (Kettner et al., 1997; Boucheny et al., 2005; Ros et al., 2006).
- LTP produced a fixed increase in synaptic efficacy each time a spike arrived through a PF to the corresponding targeted PC as defined by Equation (4). This mechanism allowed us to capture how the LTD process could be inverted when the PF stimulation was followed by spikes from the IO or by a strong depression of the Purkinje cell membrane potential (according to neurophysiologists studies; Lev-Ram et al., 2003).

The chosen mathematical-model kernel allowed accumulative computation in an event-driven simulation scheme as adopted by the EDLUT simulator (Ros et al., 2006; Luque et al., 2011a,b). This avoids the necessity of integrating the whole correlation kernel upon each new arrival of a spike. This correlation kernel, despite being computationally efficient, suffered from a second marginal peak whose impact could be considered to be negligible (<5% of the main peak height). This is indicated in the following Equation (4).

(4) $$\begin{array}{l} LTD\;.\;\Delta W_{PF_{j}-PC_{i}}\left(t\right)=\int_{-\infty }^{IO_{spike}}k\left(\frac{t-t_{IO_{spike}}}{\tau_{LTD}}\right)\;\cdot \;\delta_{GC_{spike}}\left(t\right)\;\cdot \;dt\\ \text{ if }PF_{j}\text{ is active at }t\\ LTP.\Delta W_{PF_{j}-PC_{i}}\left(t\right)=\alpha \text{ Const}.\text{ otherwise} \end{array}$$

where Δ*W*_*PFj*−*PCi*_*(t)* represents the weight change between the *j*^*th*^ PF and the target *i*^*th*^ PC. τ_*LTD*_ stands for the time constant that compensates the sensorimotor delay and δ_*GC*_ stands for the delta Dirac function defining a GC spike. For an in-depth review of the inner features of this kind of kernel (see Ros et al., 2006; Luque et al., 2011a).

#### MF–DCN synaptic plasticity

MF–DCN synaptic plasticity has been reported to depend on the intensity of the DCN cell excitation (Racine et al., 1986; Medina and Mauk, 1999; Bastian, 2006; Pugh and Raman, 2006; Zhang and Linden, 2006; Figure 2B). It has been implemented by means of a mathematical kernel defined by Equation (5):

(5) $$k\left(x\right)=e^{-\left|x\cdot \beta \right|}\cdot \cos{\left(x\right)}^{2}$$

where *x* is used as intermediate variable to get a compacted definition of the kernel, *x* is then substituted in Equation (6) by the independent variable *t*. β is a constant factor used for mitigating the impact of the second marginal peak that this kernel suffers.

(6) $$\begin{array}{l} LTD.\Delta W_{MF_{j}-DCN_{i}}\left(t\right)=\int_{-\infty }^{+\infty }k\left(\frac{t-t_{PC_{spike}}}{\sigma_{MF-DCN}}\right)\;\cdot \;\delta_{MF_{spike}}\left(t\right)\\ \;\cdot dt\text{ if }PC_{j}\text{ is active at }t\\ LTP.\Delta W_{MF_{j}-DCN_{i}}\left(t\right)=\alpha \text{ Const}.\text{ otherwise} \end{array}$$

where Δ*W*_*MFj*−*DCNi*(*t*)_ denotes the weight change between the *j*^*th*^ MF and the target *i*^*th*^ DCN, σ_*MF*−*DCN*_ stands for the window-time width of the kernel, and δ_*MF*_ stands for the delta Dirac function that defines a MF spike. As evidenced, there is no need to compensate the sensorimotor pathway delay at this plastic site since it is already compensated by the PF–PC kernel. LTD and LTP actions are then characterized as follows:

- LTD produced a synaptic efficacy decrease when a spike from the PC reached a targeted DCN. The amount of the weight decrement depended on the activity arrived through the MFs. This activity was convolved with the integrative kernel defined in Equation (5). This mainly considered those MF spikes that arrived after/before the PC–DCN spike arrival within the window-time-width defined by the kernel.
- LTP produced a fixed increase in synaptic efficacy each time a spike arrived through an MF to the corresponding targeted DCN as defined by Equation (6). This mechanism allowed the compensation of the LTD if necessary and prevents any weight saturation as proven in Luque et al. (2011a).

Despite the fact that this MF–DCN synaptic-plasticity mechanism looks very much like the mathematical expression given by PF–PC synaptic plasticity, it presents two significant differences:

- The first one lies on the reduced capability of MFs, compared to PFs, to generate sequences of non-recurrent states. As aforementioned, the MF–DCN activity compared to the analog approaches described at Garrido et al. (2013a) and Luque et al. (2014b) is now capable of codifying the passage of time. It does so by using groups of active mossy neurons that are sequentially activated. However, it uses a significantly lower number of consecutive non-recurrent time stamps than the 500 able to be generated by the granular layer (Yamazaki and Tanaka, 2007b, 2009; Yamazaki and Nagao, 2012).
  In Garrido et al. (2013a) and Luque et al. (2014b), the MF–DCN connection was implemented as a state generator able of generating only one state; the amplitude at this state was equivalent to the base current able to excite DCN cells. Plasticity at this site was capable of varying the amount of injected current that operated DCN by modifying the amount of excitation that DCN received at this connection (gain controller). However, a single-state generator was not able to generate the mentioned 500 time stamps (PF–PC). Nevertheless, having a state generator made out of clusters of non-overlapped neurons at MF–DCN allows us to roughly store or “translate” the timing sequence that is generated by a state generator holding 500 states. Given the fact that the cerebellar networks holds 2000 GCs, the simulation step-size is 2 ms, and the trajectory time is 1 s; 500 different states are, therefore, generated by groups of four non-overlapped neurons at PF–PC level. The 100 MFs have been clustered in groups of four non-overlapped neurons obtaining 25 states at MF–DCN level to roughly store the PF–PC synaptic weight distribution facilitated by those 500 different states.
- The second main difference concerns the connection driving LTD and LTP. Whilst the PF–PC plasticity was driven by the CF activity, the MF–DCN plasticity was driven by the PC activity. This mechanism optimized the activity range in the whole inhibitory pathway comprising MF–PF–PC–DCN connections: high PC activity caused MF–DCN LTD, whilst low PC activity caused MF–DCN LTP. This mechanism implemented an effective cerebellar gain controller able to adapt its output activity to minimize the amount of inhibition generated in the MF–PF–PC–DCN inhibitory loop.

#### PC–DCN synaptic plasticity

PC–DCN synaptic plasticity was reported to depend on the intensity of DCN and PC cells (Morishita and Sastry, 1996; Aizenman et al., 1998; Ouardouz and Sastry, 2000; Masuda and Amari, 2008). Moreover, plasticity at inhibitory synapses was revealed as a fundamental homeostatic mechanism in balancing the excitatory and inhibitory cell inputs (Medina and Mauk, 1999; Kleberg et al., 2014) at DCNs capable of conforming synaptic memories related to activity patterns (Vogels et al., 2011). Taking inspiration from (Medina and Mauk, 1999) and recent studies (Vogels et al., 2011; Kleberg et al., 2014), the synaptic plasticity mechanism was implemented following two possible valid kernels (Figure 2C):

- A classical inhibitory-STDP learning rule (iSTDP; Equation 7)
  (7) $$\begin{array}{l} LTP.\Delta W_{PC_{j}-DCN_{i}}\left(t\right)=e^{-\left(\frac{t_{DCN_{post}}-t_{DCN_{pre}}}{\tau_{1}}\right)}\\ \;if\;t_{DCN_{post}}>t_{DCN_{pre}}\\ LTD.\Delta W_{PC_{j}-DCN_{i}}\left(t\right)=e^{-\left(\frac{t_{DCN_{pre}}-t_{DCN_{post}}}{\tau_{2}}\right)}\\ \;if\;t_{DCN_{pre}}>t_{DCN_{post}} \end{array}$$
  where Δ*W*_*PCj*−*DCNi*(*t*)_ is the weight change between the *j*^*th*^ PC and the target *i*^*th*^ DCN. τ_1_ stands for the time constant for the LTP expression and τ_2_ stands for the time constant for the LTD expression.
- An inhibitory-STDP learning rule based on near-coincident pre and postsynaptic spikes able to potentiate inhibitory synapses, whereas every presynaptic spike causes synaptic depression (Equation 10).
  (8) $$\begin{array}{l} \Delta W_{PC_{j}-DCN_{i}}\left(t\right)=\int_{-\infty }^{+\infty }(LTP_{max}\;\cdot \;e^{-\left|\frac{t_{DCN_{post}}-t_{DCN_{pre}}}{\sigma_{PC-DCN}}\right|}\cdot \\ \;\cos{\left(\frac{t_{DCN_{post}}-t_{DCN_{pre}}}{\sigma_{PC-DCN}}\right)}^{2}-LTD_{max})\;\cdot \;dt \end{array}$$
  where Δ*W*_*PCj*−*DCNi*(*t*)_ is the weight change between the *j*^*th*^ PC and the target *i*^*th*^ DCN, σ_*PC*−*DCN*_ stands for window-time width of the kernel, and LTP_*max*_/LTP_*max*_ stand for the maximum weight depression or potentiation change per simulation step.
  These two plausible kernels mainly consider those spikes received by DCN through PC innervation within the window-time-width defined per each kernel.

### Cerebellar control loop

#### Case study A (Figure 3A)

The adopted control loop for the cerebellar architecture of Case-study-A was based on the traditional forward cerebellar control architecture. Within this architecture, the cerebellum attempted to minimize the existing difference between the controlled variable (actual cerebellar output value) and the reference variable (set point) via manipulation of the controlled variable. The reference variable (Equation 9) was a 1-s curve (2 ms time-step simulation) made out of Gaussian functions that was repeatedly iteratively presented to the cerebellar model.

(9) $$\text{reference variable}\left(t\right)=e^{-\frac{{\left(t-\frac{T}{4}\right)}^{2}}{\sigma_{ref}^{2}}}\;-\;e^{-\frac{{\left(t-\frac{3\cdot T}{4}\right)}^{2}}{\sigma_{ref}^{2}}}$$

where σ_*ref*_ stands for the Gaussian standard deviation and *T* stands for the time period.

**Figure 3:**
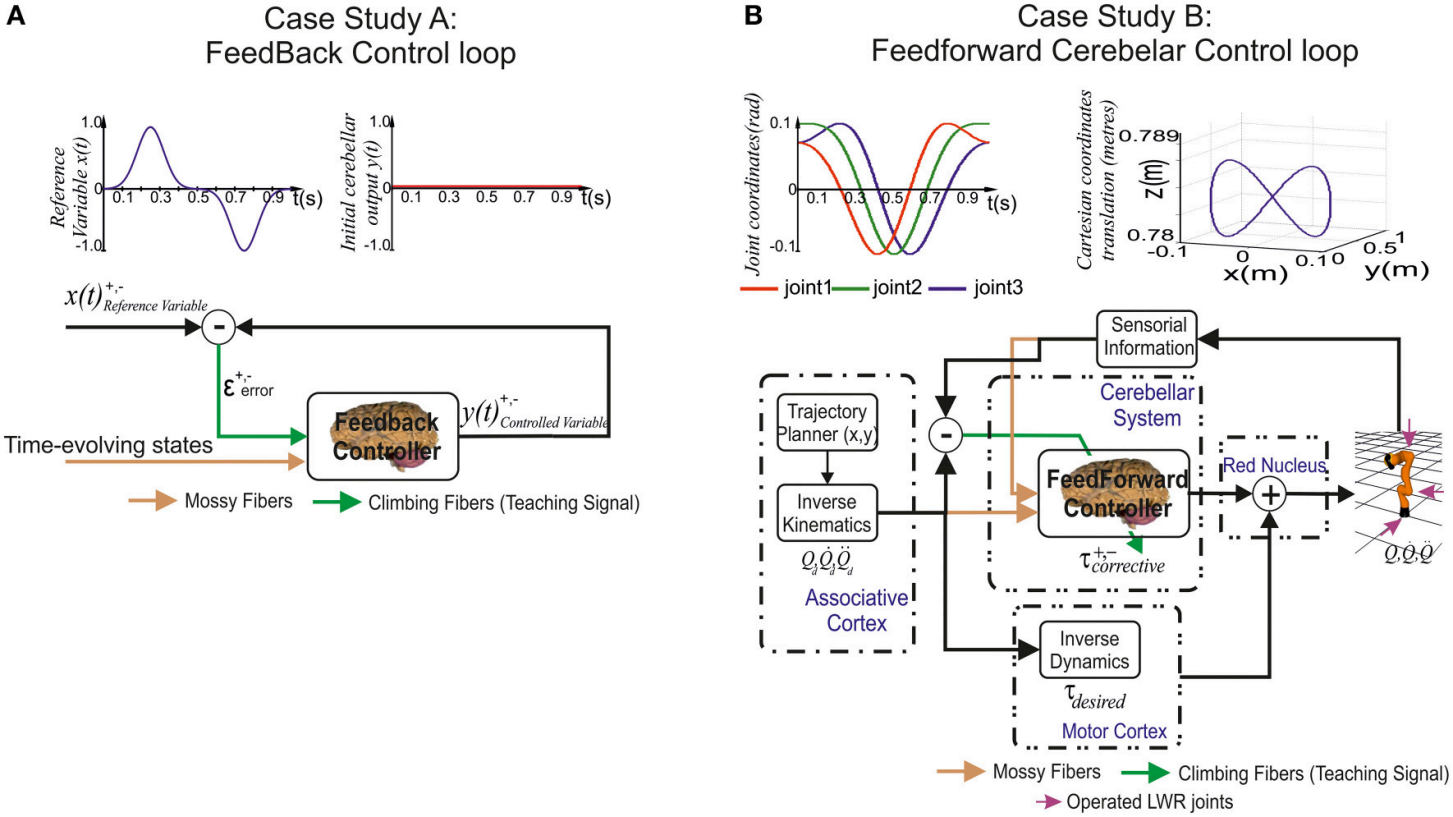
**Case study cerebellar control loops. (A)** Case study A, the adaptive cerebellar module embedded in a control loop delivers corrective actions to compensate the existing difference between a controlled variable [actual cerebellar output value *y*(*t*)] and a demanding reference variable [set point *x*(*t*)]. **(B)** Case study B, the adaptive cerebellar module embedded in a feed forward control loop delivers corrective torque values (τ_corrective_) to compensate for deviations in the crude inverse dynamic module when manipulating an object of significant weight along an eight-like trajectory. In this feed-forward control loop, the cerebellum receives a teaching error-dependent signal and the desired arm state ($Q_{d},\;{\dot{Q}}_{d},\;{\ddot{Q}}_{d}$) so as to produce the adaptive corrective actions.

This curve (Equation 9) changed its direction and module from the minimum possible value (normalized) to its maximum possible value twice per period. The cerebellar output action demanded a fine balance between the negative/positive output micro-complex actions to match the reference variable. It is worth mentioning that the IO frequency ranged between 1 and 10 Hz. Thus, according to the network already presented, each IO codified whether the error was positive or negative during 0.5 s (depending on the activated CF). Hence, no more than five spikes per IO and period (1 s) were obtained in the worst possible scenario. These directional and module changes combined with the IO biological low rate sampling constraint made the cerebellum operate at the limits of its learning performance.

#### Case study B (Figure 3B)

The adopted control loop was based on the traditional feed-forward architecture along with a crude inverse dynamic model of the simulated robotic arm. An inverse kinematic module translated the desired trajectory into arm-joint coordinates and fed an inverse dynamic module based on a recursive Newton-Euler algorithm. This algorithm generated crude step-by-step motor commands (torques) corresponding to the desired trajectory.

In light of some studies, the central nervous system has been suggested to plan and execute sequentially voluntary movements. In accordance to this hypothesis, the brain might first plan the optimal trajectory in task-space coordinates, translate them into intrinsic-body coordinates, and finally, generate the necessary motor commands (Houk et al., 1996; Nakano et al., 1999; Todorov, 2004; Hwang and Shadmehr, 2005; Izawa et al., 2012; Passot et al., 2013). According to these studies, the association cortex would be in charge of providing the desired trajectory in body coordinates and conveying them to the motor cortex which, in turn, would generate the optimal motor commands to operate our limbs. On the one hand, the spinocerebellum–magnocellular red nucleus system is thought to hold an internal neural accurate model of the musculoskeletal body dynamics learnt through sensing voluntary movements (Kawato et al., 1987). On the other hand, the cerebrocerebellum–parvocellular red nucleus system is thought to provide a crude internal neural model of the inverse-dynamics of the musculoskeletal system (Kawato et al., 1987). The crude inverse-dynamic model shall work conjointly with the dynamic model (given by the spinocerebellum–magnocellular red nucleus system) in order to get the ongoing motor commands updated to match a possible predictable error when executing a movement.

Together with the feed forward control loop, a simulated-light-weight robot (LWR) arm was integrated. The simulated-robot-plant physical characteristics can be dynamically modified to manipulate different payloads (punctual masses). This LWR (Hirzinger et al., 2000; Albu-Schäffer et al., 2007) model is a 7-DOF arm robot consisting of revolute joints where only the first (labeled as Q1), second (Q2), and fifth joint (Q3) were operated in our experiments while maintaining the others fixed (rigid).

Similarly to Case study A, the main aim when selecting a benchmark trajectory was to challenge the cerebellar learning limits. Case study B needed to reveal the dynamic properties of a simulated-robot-plant. Choosing fast movements in a smooth pursuit task consisting of vertical and horizontal sinusoidal components (Kettner et al., 1997; van Der Smagt, 2000; 1 s for the whole target trajectory) allowed us to study how inertial components (when manipulating objects) were inferred by the cerebellar architecture (Luque et al., 2014b). The selected target trajectory described an “8-shape” defined by Equation (12) in joint coordinates.

(10) $$\begin{array}{l} Q_{n}\left(t\right)=A_{n}\;\cdot \;\sin\left(\left(-4\;\cdot \;\pi \;\cdot \;t^{3}+6\;\cdot \;\pi \;\cdot \;t^{2}\right)+C_{n}\right)\\ \text{ where }n=\left\{1,\cdots ,\text{ number of links}\right\} \end{array}$$

where *A*_*n*_ and *C*_*n*_ = *n*·π/4 represent the amplitude and phase of each robot joint. The followed trajectory is based on cubic spline technique so as to provide continuity and a zero initial velocity per link, which fulfills the implementation requirements of a physical robot controller. This trajectory is easy to perform despite the non-linearity in the robot joint angles, since joint velocities and accelerations are constricted to small bounds depending on the amplitude and phase. To finally quantify and evaluate the movement performance in terms of accuracy, the average of the Mean Absolute Error (MAE) per robot joint was calculated. The estimation of this measurement was monitored in each trial, thus allowing the quantification of the global-movement accuracy evolution during the learning process.

## Results

We tested the hypothesis of cerebellar gain-controller operation assuming that the MF–DCN synaptic weights were capable of obtaining the maximum corrective cerebellar values whilst the difference between the maximum and minimum corrective cerebellar values were supplied by PC–DCN synaptic weights. We also tested the learning consolidation hypothesis by endowing these two connectivity sites with plasticity, thereby generating an internal adaptive gain controller fully compatible with the two-state learning mechanism proposed by Shadmehr and Brashers-Krug (1997), Shadmehr and Holcomb (1997), Medina and Mauk (2000), and Ohyama et al. (2006). Whilst case study A, due to its inherent simplicity, helped to demonstrate and validate our premises, case study B helped to extrapolate our premises to a more demanding scenario where the cerebellar model delivered to a simulated-robotic arm the corrective actions needed to compensate for dynamic deviations produced when manipulating heavy point masses. Furthermore, case B also helped to evaluate how the distributed learning scheme was scalable in terms of joints.

Illustrative movies of learning simulations for case study A and case study B during the manipulation of a 6-kg load are available in the Supplemental Material.

### MF–DCN STDP allows learning consolidation

In order to determine the impact of MF–DCN STDP in learning consolidation, in case-study-A, the cerebellar network was equipped with plasticity at PF–PC and MF–DCN synapses. Our first simulation was carried out to demonstrate not just, how MF–DCN could implement a gain-controller, but also how the PF–PC learning was transferred into MF–DCN synapses.

**Figure 4:**
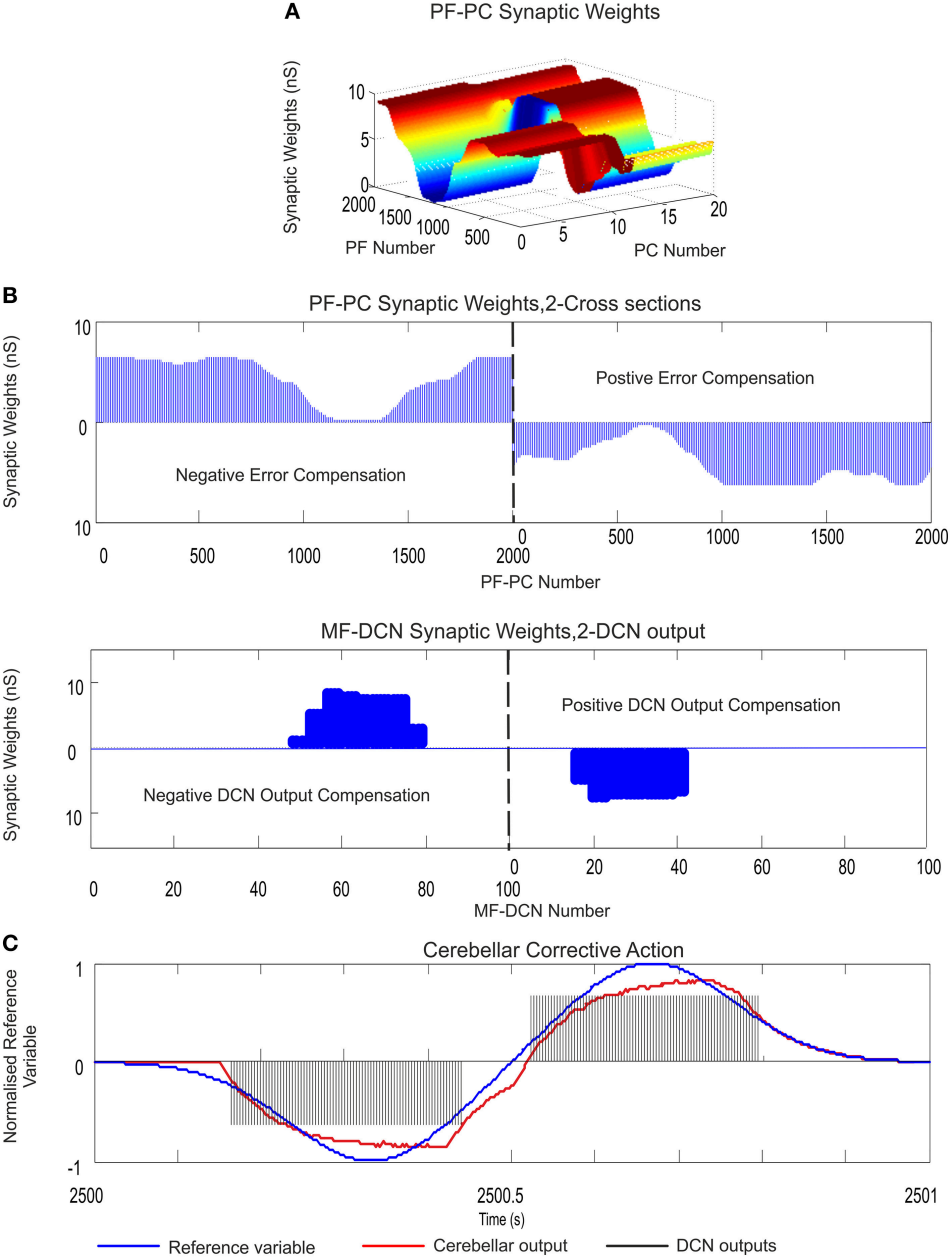
**PF–PC and MF–DCN learning rule interplay: learning consolidation. Case study A**. PF–PC and MF–DCN synaptic weight distribution at the end of the learning process. The cerebellum tries to counterbalance the existing mismatch between the actual cerebellar output and the reference variable; a 1-s curve made out of Gaussian functions which is iteratively presented to the cerebellum over 2500 iterations. The exponential weight distribution at PF–PC shows that the corrective action is properly stored at these afferents. Learning rule at PC–DCN connections is deactivated. Synaptic weights are fixed with pre-calculated values ensuring a proper inhibitory PC–DCN action. **(A)** Two micro-complexes that are conformed by 10 PCs each and innervated, in turn, by 2000 PFs. These micro-complexes are responsible for the correct balance between the negative and the positive cerebellar correction. One micro-complex is in charge of delivering the positive corrective action whilst the other one delivers the negative corrective action. Each of the two output DCN cells is, in turn, innervated by one of the two micro-complexes. **(B)** The Gaussian-like weight distribution at PF–PC is transferred in counter phase to MF–DCN synapses. The reduced MF number of non-recurrent states enables learning consolidation; however, the obtained synaptic weight-distribution shape adopts a discretized version of the PF–PC weight-distribution shape. **(C)** The injected error is properly counterbalanced thanks to the action of these two learning laws. DCN output activity (spikes in black) is transformed into its proportional analog value (in red) and, later on, subtracted from the reference variable (in blue).

Within the feed-forward control loop, the cerebellum in case study A, attempted to minimize the existing difference between the controlled variable (current cerebellar output) and the reference variable (following the 1-s curve made out of Gaussian functions; Figure 4C). The reference variable was iteratively presented over 2500 iterations. PF–PC synaptic conductances were set to an initial value of 5 nS, MF–DCN initial conditions started from zero, and PC–DCN synaptic weights were fixed with pre-calculated values that ensured a proper inhibitory PC–DCN action. In order to better discern the synaptic weight distribution shape that was transferred from PF–PC synapses into MF–DCN, the initial synaptic weights at those synaptic sites were set to equal values. This set-up configuration facilitated the perception at a glance of a continuous surface representing PF–PC synaptic distribution copying the reference variable. We also made simulations with random initialization of synaptic weights leading us to similar results but in these simulations, it was difficult to obtain a visual verification of the learning consolidation process (see Supplementary Material).

As evidenced, the reference variable changed its direction and module from the minimum possible value (normalized) to its maximum possible value twice each period, which required a fine balance between the cerebellar micro-complex negative/positive output (Figure 4C). Despite this demanding scenario, after the synaptic weight adaptation process at PF–PC connections (Figures 4A,B), the Gaussian shape that the reference variable presented, was copied and stored at PF–PC synaptic weights, thus constituting the first learning stage needed to deliver the cerebellar corrective action. Then, PF–PC learning triggered MF–DCN learning process which was able to lead MF–DCN synaptic weights to their local maximum values allowing plasticity to store temporally correlated information (the weight distribution at PF–PC was inversely copied at MF–DCN; Figure 4D).

The constrained capability of MFs, compared to PFs, when generating sequences of non-recurrent states was immediately reflected in the MF–DCN synaptic weight shape (Figure 4B). Although the reduced MF number of non-recurrent states enabled learning consolidation, the obtained synaptic weight-distribution shape, through the adaptation process at this site, was forced to adopt an inverse discretized version of the PF–PC weight-distribution shape.

### MF–DCN and PC–DCN STDP interplay toward adaptive gain controller

In order to evaluate the existing interplay amongst different forms of plasticity at PF–PC, MF–DCN, and PC–DCN synapses, respectively, case-study-A cerebellar network was sequentially added with the aforementioned adaptive mechanisms (Equations 3, 5, and 7). In previous works, we demonstrated that plasticity at PF–PC synapses could not account for preventing PC activity saturation *per se* (Garrido et al., 2013a; Luque et al., 2014b). To circumvent this limitation, MF–DCN and PC–DCN plasticity mechanisms were implemented, thus allowing PC activity to keep on operating within its optimal working range. Nevertheless, how such analog plasticity mechanisms would be re-designed and counterbalanced to take into account the spiking cerebellar nature remained an open issue.

**Figure 5:**
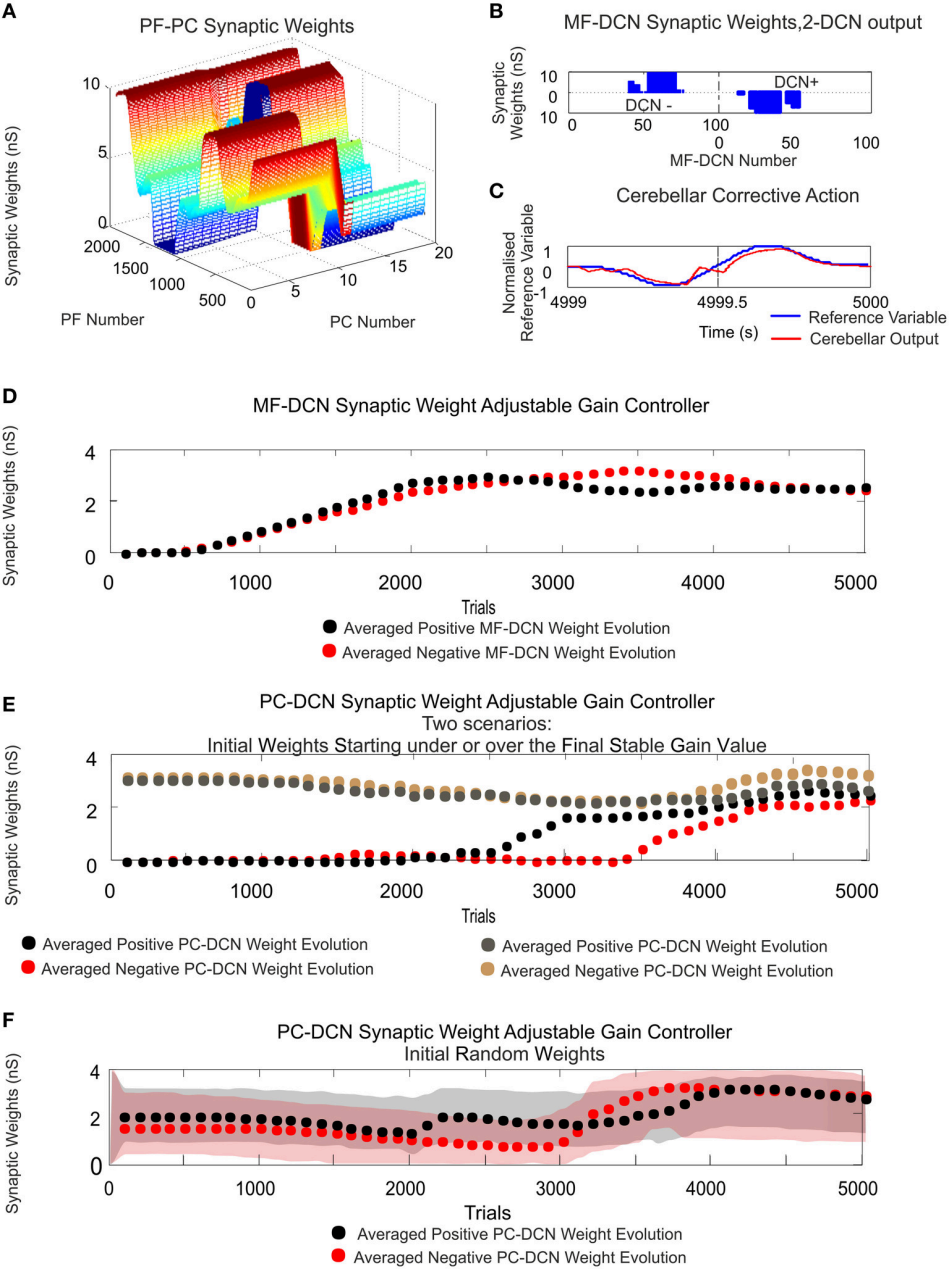
**MF–DCN and PC–DCN STDP learning rules working conjointly as an adjustable gain controller**. Case study A. **(A)** PF–PC synaptic weight distribution at the end of the learning process. The cerebellum counterbalances the existing difference between the actual cerebellar output and the reference curve which is iteratively presented to the cerebellum over 5000 iterations. The Gaussian-like weight distribution at PF–PC synapses shows that the corrective action is properly stored at these afferents. **(B)** Two micro-complexes that are conformed by 10 PCs each and innervated, in turn, by 2000 PFs are responsible for managing the trade-off between the negative and the positive cerebellar corrective action. The Gaussian-like weight distribution at PFs is inversely transferred at MF–DCN synapses. **(C)** The reference curve acting as an error is counterbalanced. DCN output activity is transformed into its proportional analog value (in red) and, later on, subtracted from the reference variable (in blue). **(D,E)** The initial conditions established for synaptic weights at MF–DCN start from zero whilst PC–DCN innervations start from either a higher or lower value than needed. MF–DCN and PC–DCN averaged synaptic weights (averaged gains) get stabilized more slowly than those at PF–PC synapses, since learning at MF–DCN and PC–DCN synapses depended on the PC activity. MF–DCN and PC–DCN averaged synaptic weights (averaged gains) are modified when PF–PC weights tend to be saturated. This learning process at MF–DCN and PC–DCN connection can be split into two components with time-constants of 750–2500 trials and 2500–5000 trials, respectively. **(F)** The initial conditions established for synaptic weights at MF–DCN start from zero whilst PC–DCN innervations start from random values. Red and gray shaded areas delimit the synaptic weight space in which the synaptic PC–DCN synaptic values evolve during the learning process for each micro-complex. Dotted lines indicate the averaged PC–DCN synaptic weight value obtained per micro-complex. The learning rule at PC–DCN self-regulates the synaptic weights obtaining the optimal firing rate demanded by both DCN and PC.

This has motivated the work presented here. Case-study-A cerebellar network attempted to minimize the existing difference between the controlled variable and the reference variable (1 s duration) over 5000 iterations (Figure 5C). PF–PC synaptic conductances were set to an initial value of 5 nS, MF–DCN initial conditions started from zero (Figure 5D), and PC–DCN synaptic weights were set to either zero initial values, random values, or higher values than needed (Figures 5E,F). As expected, the STDP learning rule located at this site was able to self-regulate PC-DCN synaptic weights in order to adequate the optimal working range demanded by both DCN and PC (Figures 5E,F).

Whilst the consolidation process was settling down (what was learnt at PF–PC synapses (Figure 5A) was transferred in counter phase to MF–DCN synapses; Figure 5B), it was possible to verify the double-learning time-scale behavior already indicated in recent behavioral and computational studies (Shadmehr and Brashers-Krug, 1997; Shadmehr and Holcomb, 1997; Medina and Mauk, 2000; Ohyama et al., 2006; Garrido et al., 2013a; Luque et al., 2014b; Movies S1, S2 in Supplementary Material). MF–DCN and PC–DCN averaged synaptic weights (averaged gains) stabilized slower than those at PF–PC synapses, since learning at MF–DCN and PC–DCN synapses depended on the PC activity. As shown in Movies S1, S2, there was a fast learning process, in which temporal information was inferred and stored at PF–PC synapses. Meanwhile, there also was a slow learning process, in which the adaptation of cerebellar excitatory and inhibitory gain values in the DCN took place. This second slow learning process could be, in turn, split into two components related to the MF–DCN and PF–PC connections with time-constants of 750–2500 trials and 2500–5000 trials, respectively. Figures 5D,E.

### iSTDP shape impacts on PC–DCN synapses

Within the case-study-A cerebellar configuration, PC–DCN iSTDP remains as the only inhibitory pathway to the cerebellar nuclei, and therefore, the only mechanism capable of reducing the cerebellar output and preventing MF–DCN from saturation. iSTDP is known to act as a fundamental mechanism in both; balancing the excitatory and inhibitory DCN inputs (Medina and Mauk, 1999; Kleberg et al., 2014), and conforming synaptic memories related to activity patterns (Vogels et al., 2011). Nevertheless, the shape held by the iSTDP at PC–DCN synapses is not yet well-known (the exact adaptation mechanism remains an open issue).

In order to identify the influence that the iSTDP shape may exert on the cerebellar output, two biologically plausible learning kernels were tested. The first one was implemented following the traditional STDP Hebbian kernel shape (Equation 7) whereas the second one was implemented following Medina and Mauk approach (Medina and Mauk, 1999), also adopted in recent studies (Vogels et al., 2011; Kleberg et al., 2014; Equation 10). The first step that needed to be proven was the robustness of the shape of these two kernels. Based on Vogels et al. (2011), these two kernels suited well our experimental test-bench, since both fulfilled two main conditions: the postsynaptic activity potentiated the activated inhibitory synapses together with the fact that in absence of postsynaptic firing, the inhibitory synapses decayed.

**Figure 6:**
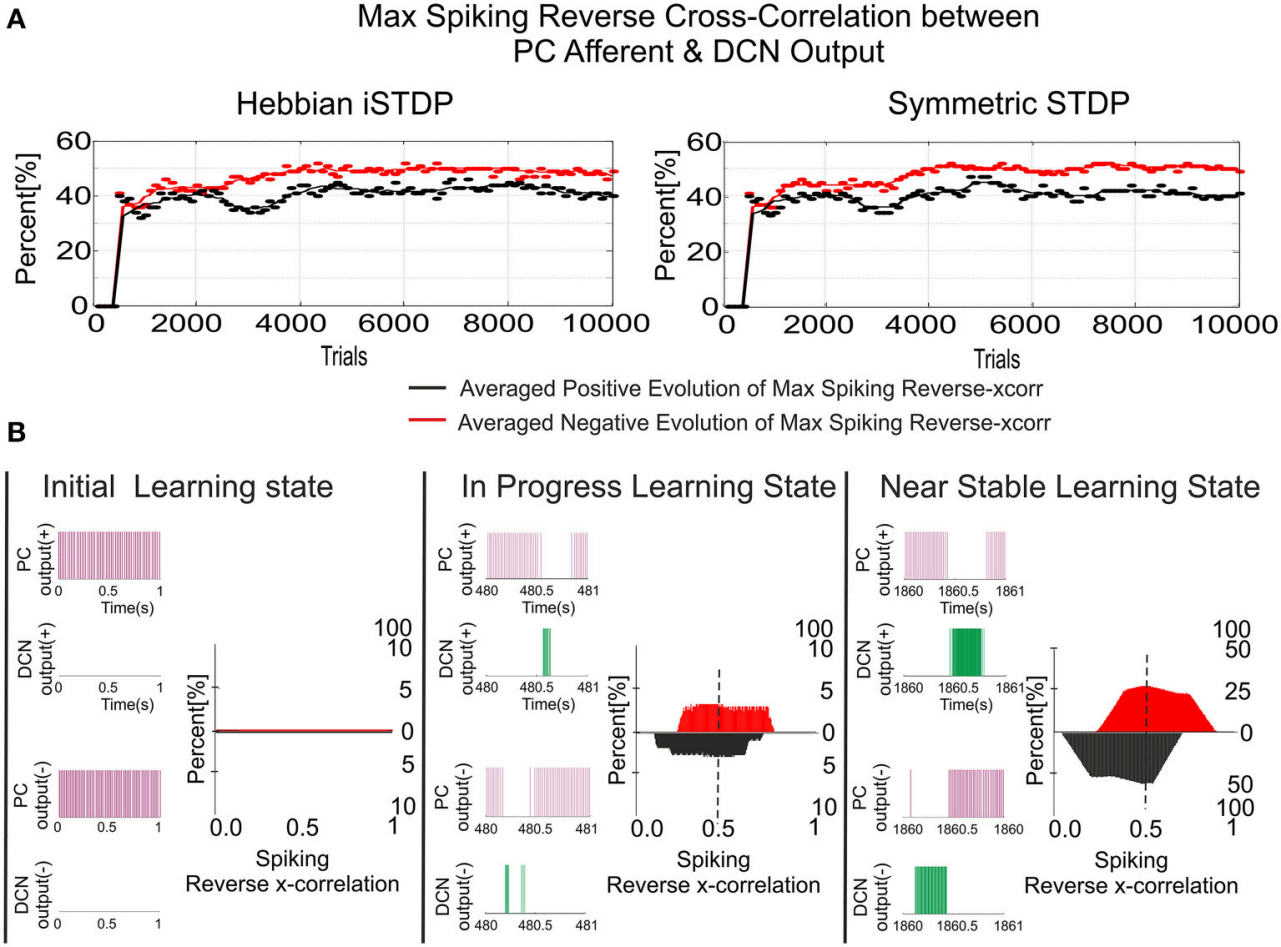
**Inhibitory plasticity at PC–DCN synapses with two possible STDP kernels, Classic i-STDP Hebbian rule, or Symmetric i-STDP**. Case study A. **(A)** Reverse cross-correlation evolution at PC–DCN synapses. Since the postsynaptic activity potentiates the activated inhibitory synapses and the inhibitory synapses decay in the absence of postsynaptic activity in both kernels, they obtain a similar performance in terms of maximal reverse correlation values and convergence speed (Vogels et al., 2011). **(B)** Distribution of the reverse cross-correlation between spike trains from PC output and DCN output. For the sake of simplicity, only the Classic i-STDP Hebbian rule is shown. Three consecutive snapshots of the DCN input/output activity are shown, where the way in which a PC is able to modulate the DCN activity can be seen.

**Figure 7:**
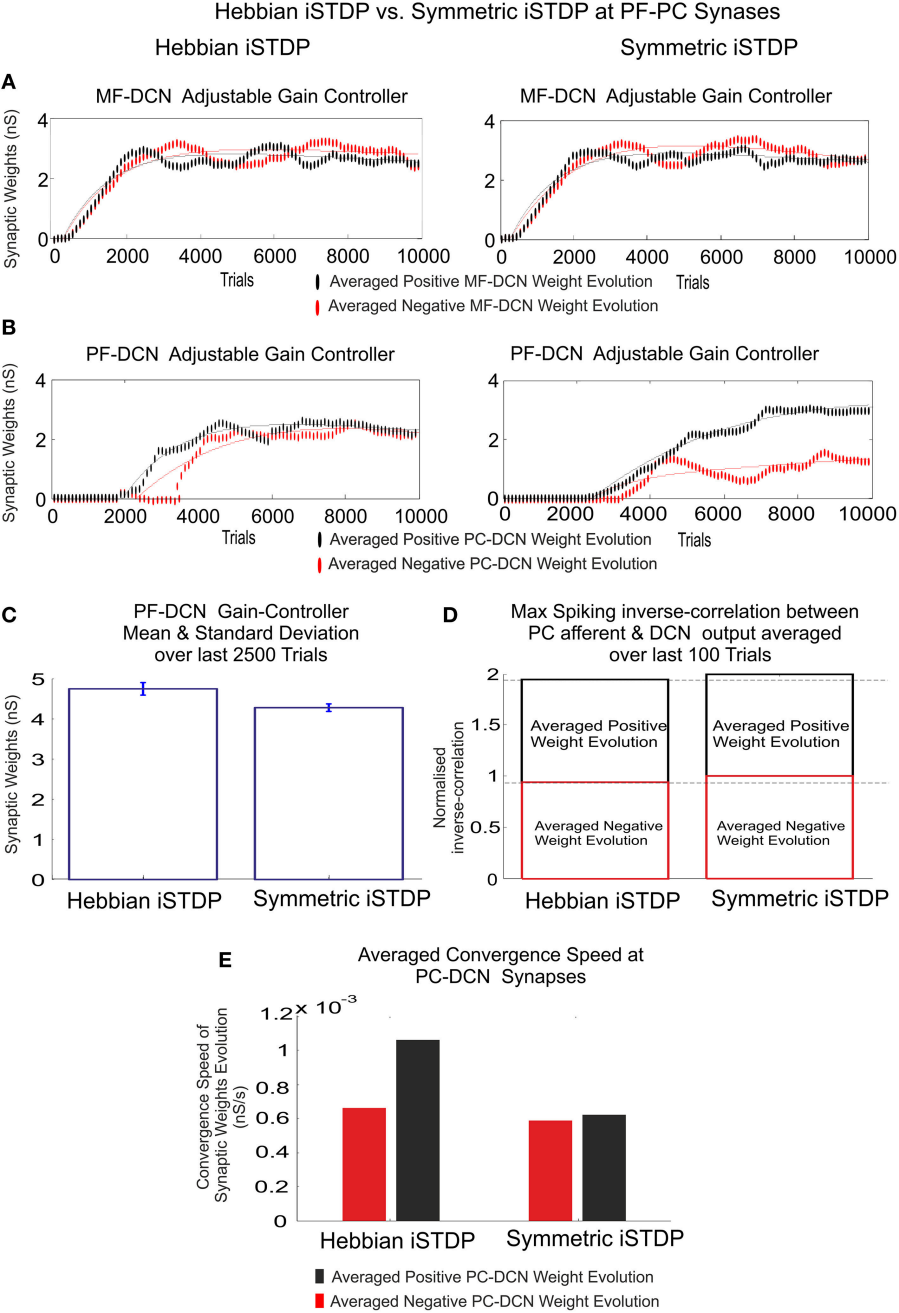
**Inhibitory plasticity at PC–DCN synapses**. Kernel shape impact. Classic i-STDP Hebbian rule vs. Symmetric i-STDP. Case study A. **(A,B)** Whilst MF–DCN averaged synaptic weights (averaged gains) exhibit a similar behavior in both configurations (Classic i-STDP Hebbian and Symmetric i-STDP), PC–DCN averaged synaptic weights (averaged gains) differ. **(C)** The second kernel presents a better performance in terms of gain stability and global gain (a lower global gain value obtains the same correction action). **(D)** The symmetric i-STDP kernel achieves a better balance for the DCN excitation and inhibition inputs. Symmetric i-STDP better propagates selectively the correlated spiking activity from PC to DCN. Symmetric i-STDP always leads to higher maximal-spiking reverse-correlation values between PC afferent and DCN output for the two DCN cells. **(E)** Averaged Convergence Speed for Classic i-STDP Hebbian kernel is higher than Symmetric i-STDP. Hebbian kernel converges faster at the cost of a lower gain stability and global gain.

Case-study-A was used for a comparative study of both approaches. Again, the cerebellar network attempted to minimize the existing difference between the controlled variable and the reference variable (1-s duration) over 10,000 iterations. PF–PC synaptic conductances were set to an initial value of 5 nS, MF–DCN initial conditions started from zero (**Figure 7A**), and PC–DCN synaptic weights were set to a zero initial value as well (**Figure 7B**). As expected, according to the aforementioned premises, both kernels showed a similar ability to correlate (more concretely, to reverse-correlate) the activity arriving from PCs with DCN output activity (Figure 6B). Both kernels did indeed obtain a similar behavior in terms of maximal reverse-correlation values and speed of convergence (Figure 6A).

Nevertheless, the second kernel exhibited a better performance in terms of stability and overall gain value (Figure 7C) but at the cost of a lower convergence speed (Figure 7E). Due to the initial conditions for DCN innervations were set initially to zero, a post-synaptic spike scenario dominated during the learning process, thus making the Hebbian approach faster than the symmetric kernel in terms of convergence speed (see Figure 2C). STDP Hebbian kernel shape has been traditionally used for spatiotemporal detection and learning of hidden spike patterns from a neural activity background by correlating post-synaptic and pre-synaptic activity (Masquelier et al., 2009). However, an inhibitory-STDP learning kernel based on near-coincident pre and post-synaptic spike seemed to be more useful for balancing the DCN excitation and inhibition inputs (Figures 7B,C) and for selectively propagating the correlated spiking activity from PC to DCN (Figure 7D).

### Testing distributed cerebellar plasticity in a robotic manipulation task

In order to quantify and extrapolate the aforementioned STDP distributed plasticity features, case-study B cerebellar network was faced with a more demanding scenario. Our last simulation was intended to show how the self-regulation of MF–DCN and PC–DCN synapses by means of STDP learning rules is able to deliver to a simulated-robotic arm the corrective actions needed to compensate for dynamic deviations produced when manipulating heavy point masses (6 kg).

**Figure 8:**
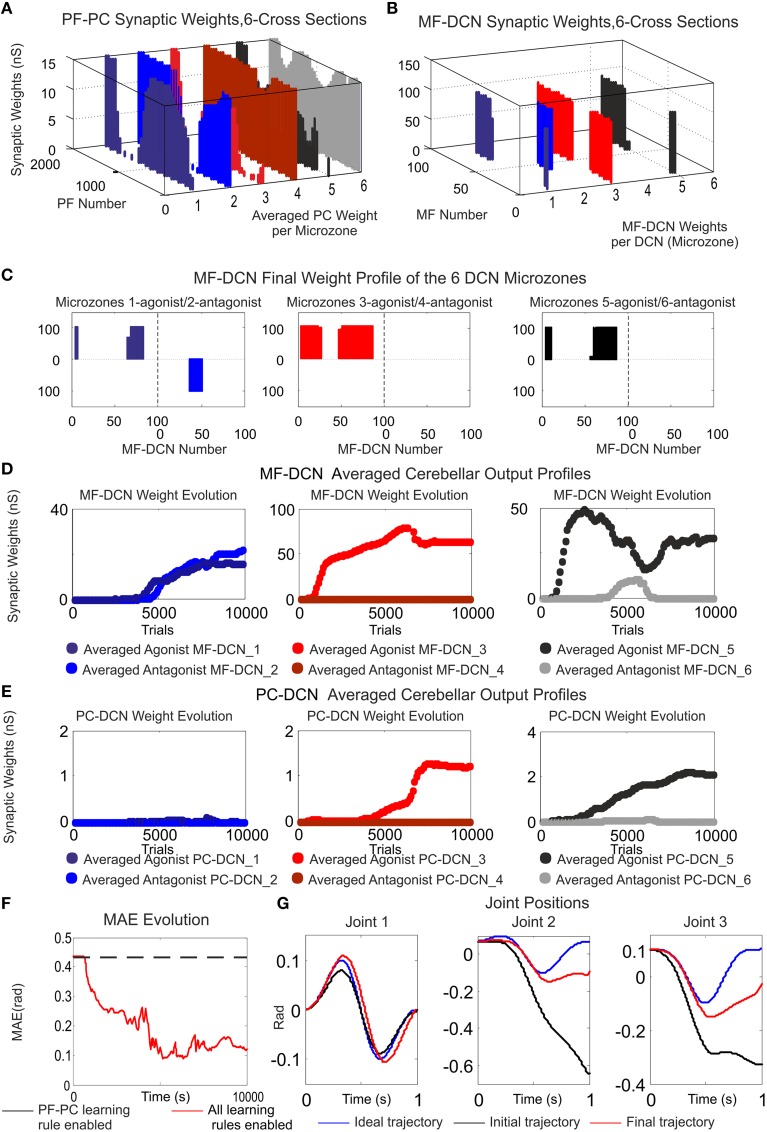
**Functionality of PF–PC, MF–DCN, and PC–DCN learning rules working conjointly in a manipulating robotic task**. Case B. **(A)** Cross-sections of the synaptic weight distribution surface at PF–PC connections at the end of the learning process. There are two cross-sections per articulated robotic joint; purple and blue cross-sections correspond to the first joint, red and orange to the second joint, and black and gray to the third one. The weight distribution at PF–PC shows that the corrective action is properly stored at these afferents. Each of the six micro-complexes is formed by 10 PCs and innervated, in turn; by 2000 PFs. **(B,C)** Each pair of micro-complexes is in charge for the correct balance between the negative and the positive cerebellar correction per each operated robot joint. One micro-complex of each pair delivers the positive corrective action per joint whilst the other one delivers the negative corrective action per joint. The weight distribution at PF–PC is transferred in counter phase to MF–DCN. Despite the reduced MF number of non-recurrent states, MF–DCN synapses are able to consolidate the learning. The obtained synaptic weight-distribution shape adopts a discretized version of the PF–PC weight-distribution shape. Since the error to be corrected at the second and third joint is unidirectional (always negative), only the antagonist correction action is delivered. **(D,E)** MF–DCN and PC–DCN averaged synaptic weights (averaged gains) are stabilized more slowly than those at PF–PC synapses. MF–DCN and PC–DCN synapses depended on PC activity and are modified when some PF–PC weights tend to be saturated. Since PCs corresponding to the first joint are operating in their proper range, there is almost no need for gain regulation (not the case of consolidation) **(F)** Medium Average Error (MAE) evolution. The error curve to be corrected is the difference between controlled (robot actual position and velocities) and reference variables (desired position and velocities Figure 3B). Controlled and reference variables are not directly related (or in the same; representation space) to cerebellar corrective actions since corrective actions are delivered in torque values whilst controlled and reference variables are taken in joint values. Despite this demanding scenario, the cerebellum is able to supply corrective torque values which decrease the error up to 77, 8% in a 6 kg manipulation task (almost max robot load) when all the learning rules are active. **(G)** Robotic joint angle corrections obtained at the end of the learning process.

Within the feed forward control architecture presented by case-study-B, the cerebellum attempted to minimize the existing difference between the controlled variable (current cerebellar output) and the reference variable (1 s eight-like trajectory to be followed by the robotic manipulator) during a manipulation task repeated 10,000 trials (Figure 8). PF–PC synaptic conductances were set to an initial value of 5 nS, MF–DCN initial conditions started from zero (Figure 8D), and PC–DCN synaptic weights were set to a zero initial value as well (Figure 8E). After DCN synaptic weight adaptation (Figures 8D,E), the cerebellum was able to deliver proper corrective torques reducing the error of the robot-arm movement (Figures 8F,G). Once the synaptic weights were stabilized, both PC and DCN neurons exploited their dynamic gain adaptation range (Figures 8D,E) allowing the cerebellum to operate near its optimal performance.

The cerebellum exhibited its ability to act as both an adaptive gain-controller (Figures 8D,E) and a distributed-learning storage architecture (what was learned at PF–PC synapses (Figure 8A) was then transferred in counter phase to MF–DCN synapses; Figures 8B,C). However, the difference between controlled and reference variable was not directly related because the cerebellar corrective action was delivered in torque commands (Figure 3B) and the proprioception state estimations were acquired in joint-angle coordinates.

It should be clarified that the proposed STDP mechanisms, and therefore their involvements, are not restricted to any specific test-bed framework, and could be extrapolated to other common but simpler test-bed frameworks such as EBCC and VOR.

## Discussion

This work presents a mechanistic spiking cerebellar model endowed with several STDP learning rules located at different synaptic sites. They are tested embedded in close-loop simulations. These close-loop simulations challenge the cerebellum with two tasks with different degrees of complexity. However, the main observation regarding the learning mechanisms at DCN synapses remains valid in all of them:

- Plasticity at DCN synapses is double-folded:
  - It is able to operate as a gain adaptation mechanism allowing the PFs to prevent saturation, thus making the learning mechanisms between PFs and PCs more accurate (keeping their plasticity capability within their working range).
  - DCN has also proven to be fundamental for the slow memory consolidation process. A plausible two-state learning mechanism (Shadmehr and Brashers-Krug, 1997; Shadmehr and Holcomb, 1997) based on STDP has been shown. According to several evidences (Shadmehr and Brashers-Krug, 1997; Shadmehr and Holcomb, 1997; Medina and Mauk, 2000; Ohyama et al., 2006), the cerebellar cortex seems to undergo a fast learning process at initial learning stages while the consolidation process seems to occur in deeper structures (more likely at DCN innervations).
- ii Inhibitory-STDP learning kernel based on near-coincident pre and post-synaptic spike has proven to be rather efficient for balancing the DCN excitation and inhibition inputs and for selectively propagating the correlated spiking activity from PCs to DCN. Nevertheless, it has been shown that the shape of the learning kernel at this site (as concluded also in Vogels et al., 2011) remains valid upon two related conditions:
  - Postsynaptic activity shall potentiate those activated inhibitory synapses.
  - In absence of postsynaptic firing, the inhibitory synapses shall decay.

### Biological realism and model limitations

Some simplifications and assumptions have been made to generate a mathematically tractable cerebellar model that is biologically realistic as well. The limitations imposed were profusely discussed in Garrido et al. (2013a) and Luque et al. (2014b); however, in light of new spiking features held by our approach, those limitations are here revisited:

- The main assumption at granular layer level is its functionality as a state generator. The state generator model is grounded in neurophysiologic observations of granule cell connectivity. Granule cells are comprised in a recurrent inhibitory network with Golgi cells, thus pointing to the fact that the input layer of the cerebellum may act as a recurrent circuit. The state-generator model has revealed that modeled granule cells present a randomly repetitive behavior in active/inactive state transitions (Yamazaki and Tanaka, 2009). Furthermore, this model has also shown that the sparse population of active cells changes with the passage of the time (POT) and no recurrence of active cell populations is exhibited. Consequently, a specific time interval can be univocally represented by means of a sequence of active cells belonging to a certain population. In other words, the state-generator model is able to represent the POT by means of a sparse-population coding scheme, thus allowing the cerebellum to operate like a liquid state machine (LSM; Maass et al., 2002; Yamazaki and Tanaka, 2007a) or an Echo state network (Jaeger, 2007). The cerebellar granule cell layer can be seen as an LSM; each LSM neuron receives time varying inputs from external sources (as the cerebellum receives varying sensorimotor inputs through mossy fibers) and from other neurons as well (this role is played in the cerebellum by different interneurons such as Golgi cells, Lugaro cells, unipolar brush cells, etc.). These LSM neurons are randomly connected to each other (as Granule cells are interconnected via Golgi cells in a recurrent loop). This structural analogy leads us to think that the recurrent nature of both neural networks, cerebellar granule layer and LSM, may operate in a similar manner. That is, the time varying inputs are turned into spatio-temporal patterns of neural activations; the granular layer acts as the reservoir of interacting spiking neurons within a recurrent topology, whilst Purkinje cells act as readout neurons. The strength of the cerebellum acting like an LSM lies in the possibility of obtaining whichever needed mathematical operation so as to perform a certain task such as eyelid conditioning or motor control tasks.
  Since the exact function of the granular layer is not fully resolved, an assessment of its involvements remains to be established besides a biologically precise representation of plasticity mechanisms underneath (i.e., Solinas et al., 2010) that could substantially modify the core conclusion of this model.
- MF input layer was assumed to maintain not only a constant firing rate, but also time-evolving states simultaneously (25 different states with four non-overlapped MFs activated per state). Making use of time-evolving states at MF layer level has, within this article, proven to be vital for the learning consolidation process. Despite this, it was assumed that the granular layer circuit was also capable of generating time-evolving states even in the presence of a constant MF input thanks to its inner dynamics (Fujita, 1982; Yamazaki and Tanaka, 2007a). DCN activity has, indeed, been traditionally related with both the excitatory-activity integration coming from MFs and the inhibitory-activity integration from PCs. The number of MFs and CFs in comparison to granule cells (GCs) is very low. Thus, these fibers (MFs and CFs) are very limited for generating a sparse representation of different cerebellar states. In fact, even though MFs in our model were able to generate 25 different states, their role could be understood more as a baseline global activity or bias term provider per generated state rather than a proper state generator that is more the role of the granular-cell-layer. This fact pointed out that the reported synaptic plasticity at MF–DCN synapses (Racine et al., 1986; Medina and Mauk, 1999; Pugh and Raman, 2006; Zhang and Linden, 2006) could induce the adjustment of gain control through plasticity at DCN synapses whilst the learning consolidation was roughly preserved at these MF–DCN synapses.
- Cerebellar feedback is needed to minimize the existing difference between the controlled variable (actual cerebellar output value) and the reference variable (set point) via manipulation of the controlled variable. We assumed the teaching signal to come only through the CFs; however, there is no general agreement regarding neither the type of information conveyed by CFs nor their potential role (Ito, 2013; Luque et al., 2014b). Furthermore, there exist evidences pointing to the fact that cerebellar feedback is bounced back toward the motor cortex (Kawato et al., 1987; Siciliano and Khatib, 2008) together with the teaching signal, which is also received and correlated at a granular layer level (Krichmar et al., 1997; Kistler and Leo van Hemmen, 1999; Anastasio, 2001; Rothganger and Anastasio, 2009). Incorporating these elements is thought to further enhance the level of flexibility and accuracy in motor control and learning.
- We have included within the model what is, to our knowledge, the most complex set of STDP plasticity mechanisms interacting with each other within the cerebellar network. Nevertheless, there are multiple sub-forms of plasticity which are still missing such as plasticity at MF–GC, GO–GC, MF–GO connections, etc., as well as PC and GC intrinsic excitability (Hansel et al., 2001; Gao et al., 2012; Garrido et al., 2013b).
- The theoretical network here presented is rather oversimplified compared to the real cerebellar network. The physiological implications may have been overlooked but must not be ignored. As an example, the role of the inhibitory PC collaterals, the complex structure of the PC dendritic tree, the operation of DCN cells with their characteristic postsynaptic rebounds, or the theta oscillations and resonance in the granular layer, amongst many other physiological evidences, shall need to be fully addressed. Nevertheless, the way in which all these physiology implications interact, how they reciprocally improve their operations, and how they are understandable in the framework of a complex cerebellar operation remains a future challenge.
- MF–DCN and PC–DCN STDP plasticity mechanisms were implemented according to some principles suggested by Medina and Mauk (2000), Masuda and Amari (2008), and Vogels et al. (2011), where DCN played the role of a further cerebellar learning vessel besides PF–PC synapses. However, the underlying mechanism that the cerebellar nuclei may experience in cerebellar learning has only been suggested at experimental single-cell level and supported by behavioral observations (EBCC and VOR). MF–DCN and PC–DCN STDP plasticity mechanisms therefore still have to be specifically demonstrated and characterized.

## Conclusion

Our results propose an explanation for the existing interplay between the excitatory and inhibitory synapses at DCN afferents by means of STDP mechanisms. This balance allows the PC outcome to shape the output of its corresponding DCN-target neuron which may effectively implement a cerebellar gain control fully compatible with the two-state learning mechanism suggested by Shadmehr and Brashers-Krug (1997), Shadmehr and Holcomb (1997), and Shadmehr and Mussa-Ivaldi (2012). Moreover, those STDP assemblies at MF–DCN and PC–DCN synapses have proven to be effective to explain how long-term memories can be transferred and stored from PF–PC to MF–DCN synapses. In fact, the experimentation revealed how MF–DCN synapses could effectively copy a discretized version of the PF–PC weight distribution shape in counter-phase. This learning consolidation process operated much as was demonstrated in Vogels et al. (2011); that is, PC, MF, or DCN cells do not compete with each other, exhibiting a winner-take-all behavior. On the contrary, the cerebellar PC–DCN, MF–DCN innervations stay inactive until PC activity starts modulating MF–DCN connections (thus favoring excitation), whilst DCN activity is able to self-modulate PC–DCN innervations (thus favoring inhibition). STDP learning rule at inhibitory synapses facilitates a self-organized balance of excitation and inhibition at DCN innervations.

Our results also suggest that the understanding of STDP mechanisms in motor learning requires not only studying their molecular basis. Rather, they show that this understanding must be accompanied by parallel insights regarding how the interactions amongst these plasticity mechanisms and the different cerebellar sub-circuitries allow distributed learning and neural homeostatic balance.

## Author contributions

All authors listed, have made substantial, direct and intellectual contribution to the work, and approved it for publication. NL and JG conceived, designed the experiments. NL performed the experiments. NL and ER analyzed the data. NL, FN, and RC contributed reagents/materials/analysis tools. NL, ER, and ED wrote the paper.

### Conflict of interest statement

The authors declare that the research was conducted in the absence of any commercial or financial relationships that could be construed as a potential conflict of interest.

## Supplementary material

Movie S1 **Learning simulation**. Synaptic weight evolution during Case A study is plotted. Simulations were run using plasticity mechanisms at PF–PC, MF-DCN, and PC-DCN along 6000 trials. PF–PC synaptic conductances were set to an initial value of 5 nS; MF-DCN and PC-DCN initial conditions started from zero. Only one every 100 trials is shown. (Top left) 3D view of the synaptic weight distribution at PF–PC synapses. (Top right) Sagittal axis of the synaptic weight distribution at MF-DCN synapses. (Second right) The cerebellum counterbalances the existing difference between the actual cerebellar output (in red) and the reference curve (in blue) which is iteratively presented to the cerebellum over 5000 iterations. (First and second plot of the bottom row). Evolution of the averaged gain at MF-DCN and PC-DCN synaptic weights at first and second micro-complexes which supply agonist (red line) or antagonist (black line) cerebellar corrective actions.

Movie S2 **Learning simulation**. Synaptic weight evolution during Case A study is plotted. Simulations were run using plasticity mechanisms at PF–PC, MF-DCN, and PC-DCN along 10,000 trials. PF–PC synaptic conductances were set to an initial value of 5 nS, MF-DCN initial conditions started from zero whilst PC-DCN initial conditions were set to a higher value than demanded. Only one every 100 trials is shown. (Top left) 3D view of the synaptic weight distribution at PF–PC synapses. (Top right) Sagittal axis of the synaptic weight distribution at MF-DCN synapses. (Second right) The cerebellum counterbalances the existing difference between the actual cerebellar output (in red) and the reference curve (in blue) which is iteratively presented to the cerebellum over 10,000 iterations. (First and second plot of the bottom row). Evolution of the averaged gain at MF-DCN and PC-DCN synaptic weights at the first and second micro-complexes which supply an agonist (red line) or antagonist (black line) cerebellar corrective action.

Movie S3 **Learning simulation**. Synaptic weight evolution during Case B study is plotted. Simulations were run using plasticity mechanisms at PF–PC, MF-DCN, and PC-DCN along 10,000 trials. PF–PC synaptic conductances were set to an initial value of 5 nS, MF-DCN and PC-DCN initial conditions started from zero. Only one every 100 trials is shown. (Top left) Sagittal axis 3D view of the synaptic weight distribution at PF–PC synapses. (Top right) Sagittal axis 3D view of the synaptic weight distribution at MF-DCN synapses. (Second row plots) Evolution of the averaged gains at MF-DCN synaptic weights from the first to sixth micro-complex. Each micro-complex supplies an agonist (red line) or antagonist (black line) cerebellar corrective action in each robot joint. The error curve to be corrected is the difference between controlled (robot actual position and velocities) and reference variables (desired position and velocities). (Third row plots) Evolution of the averaged gain at PC-DCN synaptic weights from the first to sixth micro-complexes. Each micro-complex supplies an agonist (red line) or antagonist (black line) cerebellar corrective action at its corresponding robot joint.

Figure S1 **(A)** PF–PC synaptic weight distribution at the beginning of the learning process at 1, 15, and 25 s (CASE A). The exponential weight distribution at PF–PC shows that the corrective action is properly stored at these afferents. Synaptic weights at PF–PC synapses are randomly initialized unlike in previous experimentations, where these weights were set to equal values in order to better perceive at a glance the shape of the synaptic weight distribution at this site. **(B)** MF-DCN averaged gains for 2, 3, 6, 8, and 10 kg, respectively, when the learning process has settled down (CASE B). MF-DCN synapses depended on PC activity and are modified when some PF–PC weights tend to be saturated. The heavier the payload to be manipulated by the lightweight robot, the more cerebellar gain is demanded for counterbalancing the dynamic existing mismatch between the crude inverse controller and the robot plant. Since the error is unidirectional in joints 2 and 3, the gain is unidirectional as well. In joint 1, the error to be compensated is bidirectional, and therefore, the gain has to be bidirectional.

## Appendix: neural and synapse models

Neuron models were implemented using slightly modified versions of the LIF model (Gerstner and Kistler, 2002). In the LIF model, the neural state is characterized by the membrane potential (*V*_*m*−*c*_) defined by the differential equation (Equation A1). This equation includes the effect of chemical synapses [α-amino-3-hydroxy-5-methyl-4-isoxazolepropionic acid (AMPA), gamma-aminobutyric acid (GABA) receptors] and the resting conductance (G_*rest*_),

(A1) $$\begin{array}{r} C_{m}\cdot \frac{dV_{m-c}}{dt}=g_{AMPA}\left(t\right)\cdot \left(E_{AMPA}-V_{m-c}\right)+\\ +\;g_{GABA}\left(t\right)\cdot \left(E_{GABA}-V_{m-c}\right)+G_{rest}\cdot \left(E_{rest}-V_{m-c}\right) \end{array}$$

where *C*_m_ denotes the membrane capacitance, *E*_AMPA_ and *E*_GABA_ stand for the reversal potential of each synaptic conductance, and *E*_rest_ represents the resting potential (with *G*_rest_ being the conductance responsible for the passive decay term toward the resting potential). Conductances *g*_AMPA_ and *g*_GABA_ integrate all the contributions received by each receptor type (AMPA and GABA) through individual synapses and are defined as decaying exponential functions which provide reasonable accuracy at a low computational cost (Gerstner and Kistler, 2002; Ros et al., 2006; Equation A2).

(A2) $$\begin{array}{l} g_{AMPA}\left(t\right)=\{\begin{array}{ll} 0, & \;t\leq t_{0}\\ g_{AMPA}\left(t_{0}\right)\;\cdot \;e^{-\left(t-t_{0}\right)/\tau_{AMPA}}, & \;t>t_{0}\; \end{array}\\ g_{GABA}\left(t\right)=\{\begin{array}{ll} 0, & \;t\leq t_{0}\\ g_{GABA}\left(t_{0}\right)\;\cdot \;e^{-\left(t-t_{0}\right)/\tau_{GABA}}, & \;t>t_{0}\; \end{array} \end{array}$$

where *t* represents the simulation time whilst *t*_0_ denotes the arrival instant of an input spike. *g*_*AMPA*_ stands for the AMPA receptor which provides excitation, and *g*_GABA_ stands for the GABA receptor-mediated conductance, which provides inhibition. Finally, τ_AMPA_ and τ_GABA_ are the decaying time constants of each receptor type. The parameters defining each cell type and synaptic receptor that have been chosen to model granule cell, Purkinje cell, and deep nucleus dynamics are included in Table A1.

**Table A1:** **Parameters of the cell types**.

**Parameter**	**Granule cell**	**Purkinje cell**	**DCN cell**
Refractory period (ms)	1	2	1
Membrane capacitance (pF)	2	400	2
^*^Total excitatory peak conductance	1 nS·100	1.3 nS·175,000·10%^*^	1 nS·7
Total inhibitory peak conductance	1 nS·200	3 nS·150	30 nS·1
Threshold (mV)	−40	−52	−40
Resting potential (mV)	−70	−70	−70
Resting conductance (nS)	0.2	16	0.2
Resting time constant (τ_rest_; ms)	10	25	10
Excitatory-synapse time constant (τ_AMPA_; ms)	0.5	0.5	0.5
Inhibitory-synapse time constant (τ_GABA_; ms)	10	1.6	10

Parameters obtained from the following papers:

Granule cell (GC; Silver et al., 1996; Tia et al., 1996; Nusser et al., 1997; D'Angelo et al., 1998; Rossi and Hamann, 1998) and Purkinje cell (PC; D'Angelo et al., 1993, 1998, 2001; Nieus et al., 2006). DCN data were extracted from unpublished material from Prof. D'Angelo's lab.

* Where 10% means the ratio of active connections PF–PC (out of the total 175,000 PFs).

## Abbreviations

PF: parallel fiber
MF: mossy fiber
CF: climbing fiber
GC: granule cell
GoC: Golgi cell
PC: Purkinje cell
DCN: deep cerebellar nuclei
IO: inferior olive
MLI: molecular layer interneuron
MAE: mean average error
EBCC: eye blink classical conditioning
VOR: vestibulo-ocular reflex.
