# Supplementary figures and images for: Distributed Cerebellar Motor Learning: A Spike-Timing-Dependent Plasticity Model
Source: Front Comput Neurosci. 2016 Mar 2;10:17. doi: 10.3389/fncom.2016.00017 (PMC4773604; doi:10.3389/fncom.2016.00017)

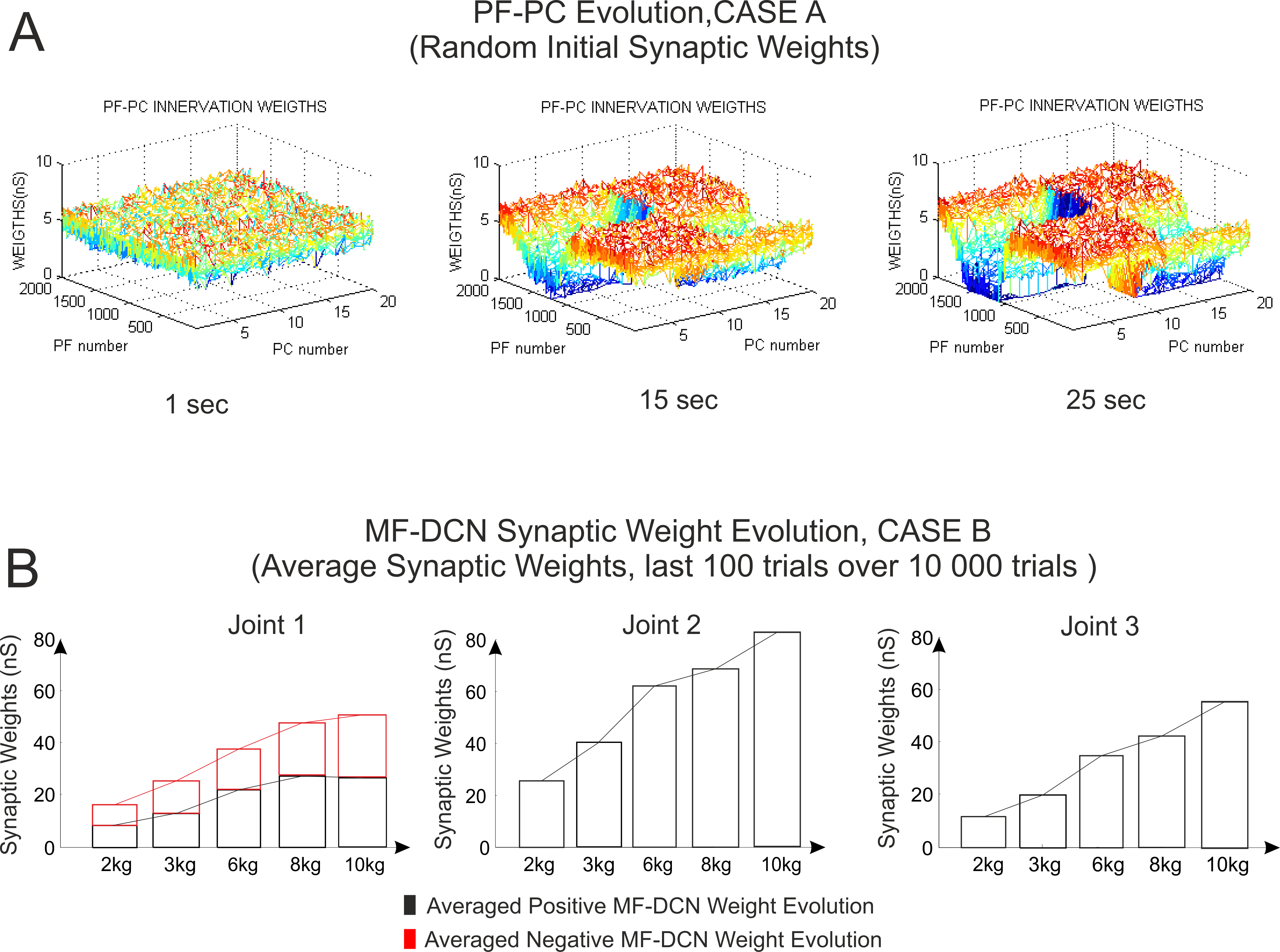

Supplement: Figure S1 — (A) PF–PC synaptic weight distribution at the beginning of the learning process at 1, 15, and 25 s (CASE A). The exponential weight distribution at PF–PC shows that the corrective action is properly stored at these afferents. Synaptic weights at PF–PC synapses are randomly initialized unlike in previous experimentations, where these weights were set to equal values in order to better perceive at a glance the shape of the synaptic weight distribution at this site. (B) MF-DCN averaged gains for 2, 3, 6, 8, and 10 kg, respectively, when the learning process has settled down (CASE B). MF-DCN synapses depended on PC activity and are modified when some PF–PC weights tend to be saturated. The heavier the payload to be manipulated by the lightweight robot, the more cerebellar gain is demanded for counterbalancing the dynamic existing mismatch between the crude inverse controller and the robot plant. Since the error is unidirectional in joints 2 and 3, the gain is unidirectional as well. In joint 1, the error to be compensated is bidirectional, and therefore, the gain has to be bidirectional. [file Image1.tif]
